# IRE1α translational suppression potentiates STING-dependent chemoresistance in pancreatic cancer

**DOI:** 10.1038/s41419-025-07999-x

**Published:** 2025-10-06

**Authors:** Yuan Luo, Mengqi Sun, Lei Chang, Zinan He, Xinghang Zhou, Yaming Yuan, Huijuan Sun, Shiqi Luo, Jinyan Huang, Hongkun Wu, Wenjun Liu, Zhangsen Zhou, Yuanhui Mao, Yewei Ji, Tingbo Liang

**Affiliations:** 1 Zhejiang Provincial Key Laboratory of Pancreatic Disease, MOE Joint International Research Laboratory of Pancreatic Diseases & Department of Hepatobiliary and Pancreatic Surgery, The First Affiliated Hospital, Zhejiang University School of Medicine, Hangzhou, China; 2 Key Laboratory of Molecular Biology for Infectious Diseases (Ministry of Education) & Department of Infectious Diseases of the Second Affiliated Hospital, Chongqing Medical University, Chongqing, China; 3 Shanghai Institute of Nutrition and Health, Chinese Academy of Sciences, Shanghai, China; 4 Department of Urology of the Second Affiliated Hospital, Liangzhu Laboratory, School of Medicine, Zhejiang University, Hangzhou, China; 5 Present Address: Department of Immunology, School of Basic Medical Science & Chongqing Key Laboratory of Tumor Immune Regulation and Immune Intervention, Chongqing Medical University, Chongqing, China

**Keywords:** Cell signalling, Cancer microenvironment

## Abstract

Chemotherapy remains a standard treatment for pancreatic ductal adenocarcinoma (PDAC); however, its effectiveness is limited, and the underlying mechanisms are poorly understood. STING plays diverse and critical roles in cancer, yet the role of PDAC cell-intrinsic STING signaling and its regulation under chemotherapy remain unclear. Here, we report that chemotherapy induces cancer cell-intrinsic STING signaling and that STING deletion in PDAC enhances cell death under chemotherapy while suppressing tumor growth in both immune-deficient and immune-competent mice. Interestingly, chemotherapy selectively inhibits translation of IRE1α, an ER membrane protein and a canonical mediator of ER stress. Loss of IRE1α in PDAC amplifies STING signaling and increases resistance to chemotherapy. Mechanistically, IRE1α interacts with STING via their transmembrane regions, reducing STING stability in PDAC cells. Our study reveals that PDAC cells downregulate IRE1α to reinforce STING-mediated pro-survival response; however, this adaptation also makes them more vulnerable to proteostasis imbalance and ER stress-induced cell death. Notably, we demonstrate that combining ER stress inducers with STING signaling inhibition enhances chemotherapy efficacy both in vitro and in vivo.

## Introduction

Pancreatic ductal adenocarcinoma (PDAC) is the most common and lethal form of pancreatic cancer, currently ranking as the fourth leading cause of cancer-related deaths worldwide, with a five-year overall survival rate of just 12% [1]. The incidence of PDAC is expected to more than triple in the next decade [2, 3]. Chemotherapy, including agents such as platinum compounds (cisplatin, oxaliplatin), fluorouracil (5-FU), gemcitabine, and albumin-bound paclitaxel, is a standard treatment modality used across various stages of PDAC, including adjuvant therapy and for patients with advanced or metastatic disease [4, 5]. However, PDAC often exhibits poor responsiveness and develops resistance to these treatments [4, 5]. Thus, there is an urgent need to delve deeper into the cellular and biochemical alterations in PDAC cells post-chemotherapy and identify novel strategies that could enhance therapeutic outcomes.

The cyclic GMP-AMP synthase (cGAS)-Stimulator of Interferon Genes (STING) signaling cascade is mediator of the innate immune response and other cellular functions in response to cytosolic double-stranded DNA (dsDNA) [6–8]. Recent studies have highlighted the critical roles of cGAS-STING activity across a spectrum of pathological processes, including infection, autoimmunity, aging, and tumor immunity [6, 9–16]. In cancer, the STING pathway plays dual role [17]. On one hand, cGAS-STING signaling in antigen-presenting cells within the tumor microenvironment enhances anti-tumor T cell response [18, 19], and STING agonists has shown promise in promoting immunogenic tumor cell death [20]. Conversely, the activation of tumor cell-intrinsic STING by cytosolic DNA, due to chromosomal instability (CIN) or DNA damage from treatment, can support cancer cell survival, tumor growth, and metastasis through mechanisms that remain partially understood [16, 21, 22]. Furthermore, STING-mediated cell death of T cells and dendritic cells (DCs) in the tumor microenvironment can impair immune response and limit the efficacy of STING agonist-based antitumor therapies [23, 24]. Given the complex and context-dependent nature of STING signaling, a deeper investigation into how cGAS-STING is activated and regulated in cancer cells under different clinical conditions is crucial. Understanding these mechanisms could help identify strategies to shift STING activity from supporting tumor survival to enhancing antitumor immunity.

The endoplasmic reticulum (ER) is a multifunctional organelle critical for protein synthesis, folding, and secretion [25–27]. The unfolded protein response (UPR), mediated by ER transmembrane sensors including inositol-requiring enzyme 1α (IRE1α), protein kinase R-like ER kinase (PERK), and activating transcription factor 6 (ATF6), plays a key role in maintaining protein homeostasis and cellular viability [28]. Disruption in protein synthesis and folding are common across various cancers, with UPR pathways significantly influencing tumor progression [29, 30]. The ER also serves as a pivotal platform for the regulation of STING functions [8]. Thus, it is important to investigate how the ER proteostasis network is modulated and how UPR pathways intersect with STING signaling in tumor cells undergoing chemotherapy.

In this study, we report that chemotherapy activates cGAS-STING signaling in PDAC cells, leading to a pro-survival inflammatory response. Notably, chemotherapy-induced STING signaling and its regulation in cancer cells diverges significantly from that triggered by DMXAA in macrophages. Our findings reveal that the UPR mediator IRE1α acts as a negative regulator of STING in PDAC cells and that PDAC cells adapt to chemotherapy by suppressing IRE1α translation, thereby enhancing STING signaling. However, this downregulation of IRE1α makes cancer cells less adaptable to induced ER stress. Exploiting this vulnerability, we demonstrate that combining ER stress inducers with STING inhibition significantly improves chemotherapy efficacy in PDAC. Our study identifies a promising therapeutic strategy that leverages the altered signaling landscape of cancer cells to enhance treatment outcomes.

## Results

### Chemotherapy drugs induce inflammation via the STING pathway in PDAC

**Fig. 1:**
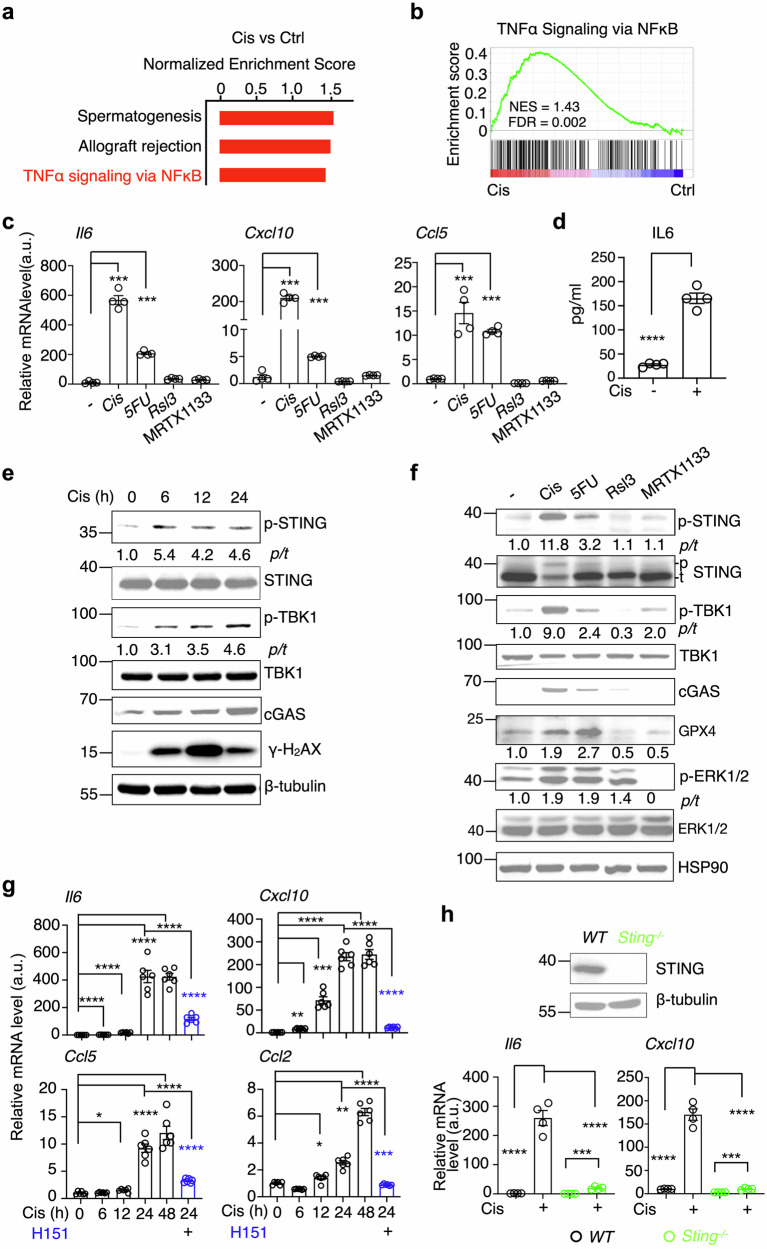
Chemotherapy drugs induce inflammation via the STING pathway. **a**, **b** GSEA of upregulated genes in KPC cells treated with cisplatin (Cis, 20 μM) for 24 hours identified enriched gene sets (n = 3). **c** q-PCR analysis of *Il6, Cxcl10*, and *Ccl5* in KPC cells treated with Cis (20 μM), 5-fluorouracil (5FU, 20 μM), ferroptosis inducer Rsl3 (20 μM), and KRAS inhibitor MRTX1133 (20 μM) for 22 hours (n = 4, representative of two independent biological repeats). **d** ELISA analysis of IL6 in culture medium from KPC cells treated with Cis (n = 4, representative of two independent biological repeats). **e** Immunoblot analysis of indicated proteins in KPC cells treated with 20 μM Cis at various time points, representative of three independent biological repeats. **f** Immunoblot analysis in KPC cells treated with Cis, 5FU, Rsl3, and MRTX1133 for 22 hours, representative of two independent biological repeats. **g** q-PCR analysis of inflammatory genes in KPC cells treated with Cis alone or in combination with H151 (4 μg/mL, pretreated 1 h prior to Cis treatment) for the indicated time (n = 6, combined from two independent biological repeats). **h** q-PCR analysis of inflammatory genes in wild-type (*WT*) or *Sting* ^-/-^ KPC cells treated with or without Cis (n = 4, representative of two independent biological repeats). Immunoblot analysis of STING protein in wild-type (*WT*) or *Sting*^-/-^ cells is shown above. The relative intensity of proteins (normalized to β-tubulin or HSP90) or the phosphorylated-to-total protein ratio (*p/t*) is shown below the blots (**e**, **f**). All values are presented as means ± SEM. Statistical significance was determined using an unpaired, two-tailed Student’s t-test, *, p < 0.05; **, p < 0.01; ***, p < 0.001; ****, p < 0.0001 (**c**, **d**, **g**, **h**).

Chemotherapy resistance remains a major obstacle in the treatment of pancreatic cancer [4, 5]. To unravel the molecular mechanisms driving tumor responses to chemotherapy in pancreatic cancer, we conducted RNA sequencing to compare the global transcriptional profiles of KPC cells, a pancreatic ductal adenocarcinoma (PDAC) cell line derived from the *Kras*^*LSL-G12D*^;*Trp53*^*LSL-R172H*^*; Pdx1-Cre* mice, following 24 hours of treatment with cisplatin (Cis), a standard chemotherapy drug, against untreated controls. Gene set enrichment analysis (GSEA) identified TNF-α signaling via the NF-κB pathway as one of the three significantly enriched pathways during chemotherapy (Fig. 1a, b). Quantitative PCR confirmed substantial upregulation of inflammatory genes, including *Il6*, *Cxcl10*, and *Ccl5*, in KPC cells post-Cis treatment (Fig. 1c). ELISA analysis found that the culture medium from KPC cells post-Cis treatment enriched with IL6 (Fig. 1d). This inflammatory response was also observed with another common chemotherapy drug, 5-fluorouracil (5FU), but was absent in cells treated with the ferroptosis inducer Rsl3 or the KRAS inhibitor MRTX1133 (Fig. 1c), indicating that the inflammation is specific to chemotherapy-induced cellular stress rather than a generalized cellular injury response.

The cGAS-STING pathway has been implicated in linking chemotherapy-induced DNA damage to inflammation [31]. Consistent with this role, we observed increased levels of cGAS protein, phosphorylation of STING, and its downstream effector TANK-binding kinase 1 (TBK1) in KPC cells post-Cis treatment (Fig. 1e), alongside increased phosphorylation of H2AX, a DNA damage marker (Fig. 1e). Parallel findings were noted in Panc02 cells, another PDAC line derived from tumors chemically induced by 3-methylcholanthrene, which showed marked increases in STING and TBK1 phosphorylation and upregulation of inflammatory genes post-Cis treatment (Fig. S1a, b). Notably, while Rsl3 and MRTX1133 treatment led to a reduction in GPX4 expression and ERK phosphorylation, respectively, they barely induced activation of the cGAS-STING pathway, which was robustly activated by chemotherapy agents (Fig. 1f), further emphasizing the link between chemotherapy and cGAS-STING activation. Chemotherapy drugs led to significant upregulation of inflammatory gene transcription as early as 6 hours, and peaking at 24-48 hours post-treatment, while the STING antagonist H151 effectively suppressed this response during the plateau phase (Fig. 1g). *Ifnb1* is a key transcriptional target of STING, however, it’s frequently under detection limit in our experimental system, indicating a variable cellular activity between tumor cell and immune cells. To further validate the role of STING in the regulation of chemotherapy-induced inflammation, we generated STING-deficient KPC and Panc02 cells using the CRISPR/Cas9 system. STING deletion resulted in a marked reduction in the expression of inflammatory genes following 24 hours of Cis treatment (Figs. 1h and S1c). Collectively, these findings underscore the pivotal role of the STING pathway in mediating chemotherapy-triggered inflammatory responses in PDAC cells.

### STING signaling promotes PDAC survival under chemotherapy

**Fig. 2:**
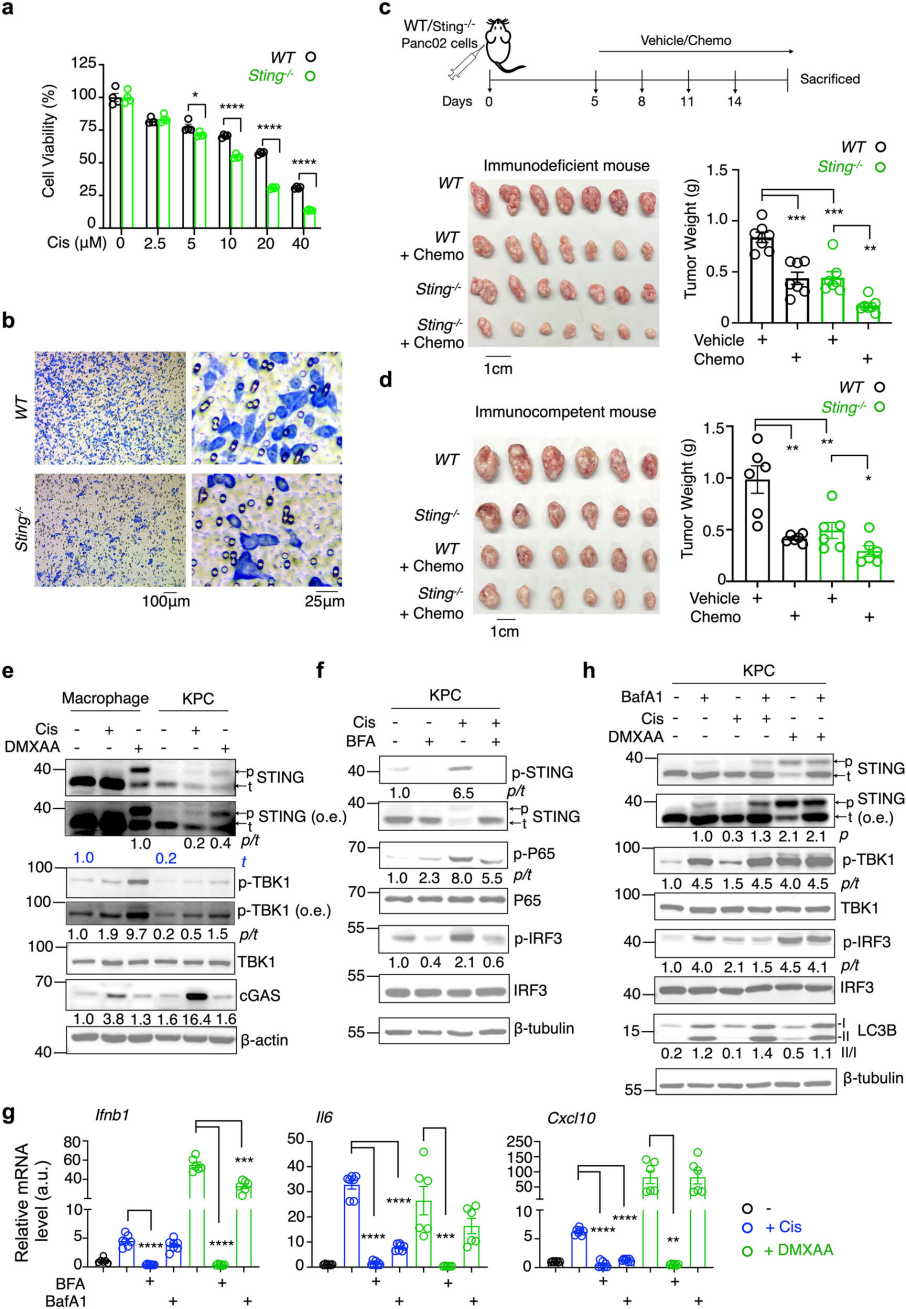
STING signaling in tumor cells promotes survival and exhibits distinct modulation compared to macrophages. **a** CCK-8-based cell viability assay comparing *WT* and *Sting* ^-/-^ Panc02 cells treated with varying Cis doses for 24 hours (n = 4, representative of two independent biological repeats). **b** Transwell migration assay of *WT* and *Sting* ^-/-^ Panc02 cells; images representative of two independent repeats. **c**, **d** Schematic representation of the experimental cancer model in which tumor-transplanted immunodeficient or immunocompetent mice received four intraperitoneal (i.p.) injections of chemotherapy drugs (Cis, 3 mg kg^−1^; 5FU, 25 mg kg^−1^; Irinotecan, 25 mg kg^−1^). Representative images of pancreatic tumors in immunodeficient (**c**) and immunocompetent (**d**) mice subcutaneously inoculated with *WT* or *Sting* ^-/-^ Panc02 cells and treated with either vehicle or chemotherapy drugs. Quantitation of tumor weights at the end of experiment shown on the right, with n = 7 mice per group for (**c**), and n = 6 mice per group for (**d**), representative of two independent biological repeats. Immunoblot analysis of STING pathway proteins in macrophages and KPC cells treated with Cis (20 μM for 24 hours) or DMXAA (20 μg/mL for 3 hours) in (**e**), or Cis, Brefeldin A (BFA,5 μg/mL), or both for 24 hours (**f**). **g** q-PCR analysis of inflammatory genes in Panc02 cells treated with Cis or DMXAA, ± BFA or BafA1 (n = 6-7, combined from two independent biological repeats). **h** Immunoblot analysis of proteins in KPC cells treated with Cis or DMXAA ± Bafilomycin A1 (BafA1, 20 nM for 3.5 hours). The relative intensity of proteins (normalized to β-actin or β-tubulin) or the phosphorylated-to-total protein ratio (*p/t*) are shown below the blots (**e**, **f**, **h**). o.e. overexposed. Values are presented as means ± SEM. Statistical significance was determined using an unpaired, two-tailed Student’s t-test, *, p < 0.05; **, p < 0.01; ***, p < 0.001; ****, p < 0.0001 (**a**, **c**, **d**, **g**).

To further elucidate the physiological role of STING signaling in PDAC, we conducted both in vitro and in vivo analyses. In vitro, Panc02 cells deficient in STING exhibited increased sensitivity to Cis and a significant reduction in migratory capacity, as evidenced by transwell migration assay (Fig. 2a, b). In vivo, after subcutaneous implantation of wildtype (WT) or *Sting* knockout (*Sting* ^-/-^) Panc02 cells into athymic Nude immunodeficient mice and immunocompetent C57BL/6 mice, *Sting* ^-/-^ cancer cells displayed markedly slower tumor growth and increased sensitivity to chemotherapy compared to control cancer cells (Fig. 2c, d), suggesting a pro-tumorigenic role of STING activation that is largely independent of T cell-mediated immunity and instead acts through tumor-intrinsic mechanisms. Furthermore, administration of the STING inhibitor H151 significantly inhibited the growth of implanted PDAC cells in Nude mice (Fig. S1d). These findings support the conclusion that tumor-intrinsic STING signaling confers a survival advantage to PDAC cells, highlighting its potential role as a target for therapeutic intervention.

### STING signaling is regulated differently in cancer cells

Given the significant role of STING in PDAC cells, we investigated the regulation of STING signaling in PDAC. While the activation of STING and its regulation are well-characterized in agonist-stimulated macrophage cells, we compared STING signaling activation by cisplatin and the STING agonist DMXAA in PDAC cells and macrophage. Notably, the total STING protein levels in PDAC cells at baseline were substantially lower than those in macrophage (Fig. 2e, lanes 1 vs. 4), despite comparable levels of other components in the pathway, including the upstream sensor cGAS and downstream effectors TBK1 (Fig. 2e, lanes 1 vs. 4). Consequently, the phosphorylation levels of STING and TBK1 in PDAC cells were significantly lower compared to macrophages when treated with DMXAA (Fig. 2e, lanes 3 vs. 6). Cis treatment induced mild yet detectable levels of phosphorylation of STING and TBK1, while significantly increasing cGAS levels in PDAC cells (Fig. 2e, lanes 4 vs. 5).

STING activation by canonical agonists induces conformational changes that facilitate its translocation from the ER to the trans-Golgi network (TGN), followed by recruitment and activation of downstream TBK1 [14, 32, 33]. Our studies showed that the ER-Golgi transport inhibitor Brefeldin A (BFA) blocked cisplatin- and DMXAA-induced phosphorylation of STING, P65, and IRF3, as well as the expression of inflammatory genes (Fig. 2f, g), suggesting that STING activation in PDAC still depends on ER exit. In innate immune cells, activated STING is trafficked from the Golgi to acidic endolysosomes for the termination of STING signaling, as disruption of lysosomal degradation amplifies STING-medicated inflammation [13, 14, 34, 35]. Unexpectedly, the inhibition of lysosomal function using Bafilomycin A1 (BafA1), which impaired autophagic cargo degradation and increased the LC3B-II/I ratio (Fig. 2h), did not amplify STING-mediated inflammatory gene expression in PDAC cells treated with Cis or DMXAA (Figs. 2g and S1e), nor did it enhance STING downstream signaling (Fig. 2h, lanes 2 vs. 4, lanes 5 vs. 6), indicating that STING signaling in PDAC is regulated distinctly from that in immune cells. Thus, while STING exits the ER in PDAC cells upon stimulation, the down-regulation of its signaling may not primarily rely on endolysosomal degradation. This suggests that regulatory pathways involving the ER and other organelles upstream of the endolysosome may play a pivotal role in controlling STING signaling in PDAC.

### Chemotherapy drugs downregulate the IRE1α branch of the ER stress network

Next, we sought to identify potential PDAC-intrinsic regulator(s) of STING. Both Cis and 5FU induce tumor cell death by causing DNA damage, and DNA damage response factors are directly involved in STING activation under etoposide-induced DNA damage in human keratinocytes [36]. To explore this further, we investigated the regulation of cGAS-STING signaling by DNA damage response (DDR) pathways in PDAC. KU-55933 and VE-821, respective inhibitors of ataxia-telangiectasia mutated kinase (ATM) and ataxia telangiectasia and Rad3-related protein (ATR), which are essential for the phosphorylating of H2AX to initiate DNA repair [37], did not inhibit Cis-induced transcription of inflammatory genes *Il6* and *Ccl5* (Fig. S1f). Thus, DDR signaling does not directly contribute to chemo-induced STING signaling in PDAC. The oncogenic HER2 pathway has been reported to suppress STING signaling [38]. We found that KRAS inhibitor MRTX1133, which targets oncogenic KRAS^G12D^, did not promote STING signaling in PDAC cells (Fig. 1c, f).

**Fig. 3:**
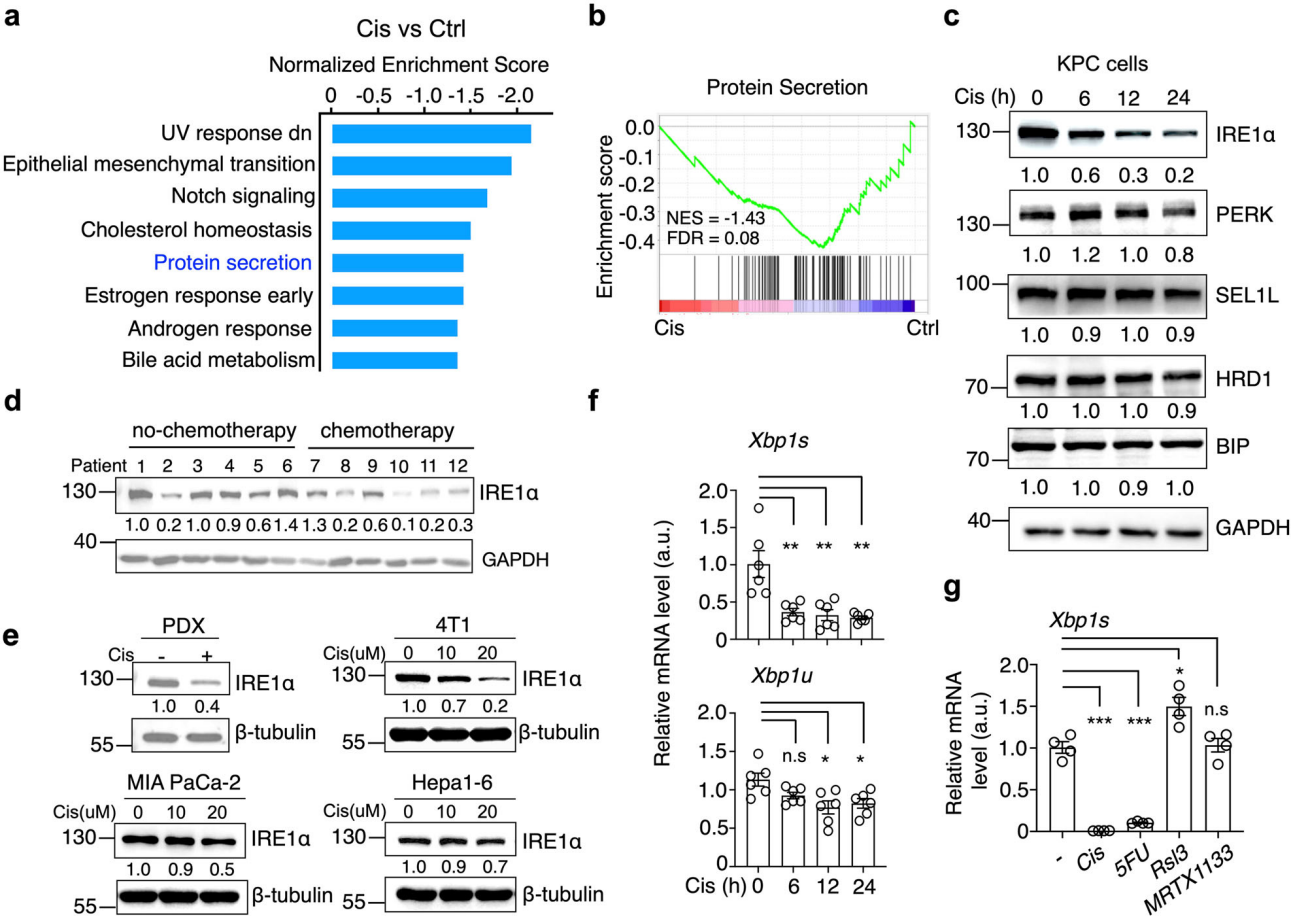
Chemotherapy-induced alterations in the ER proteostasis network in cancer cells. **a**, **b** Downregulated gene sets in KPC cells treated with 20 μM Cis for 24 hours (n = 3). **c** Immunoblot analysis of ER stress pathway proteins in KPC cells treated with Cis at various time points. Data are representative of six independent biological repeats for IRE1α protein, and three for others. The quantification of IRE1α protein levels is shown in Fig. S2a. **d** Immunoblot analysis of IRE1α protein in PDAC patient tumor samples treated with or without chemotherapy, with each lane representing a sample from one patient. **e** Immunoblot analysis of tumor cells derived from patient-derived xenograft (PDX) mice, human pancreatic tumor MIA PaCa-2 cells, mouse breast cancer 4T1 cells, and hepatoma Hepa1-6 cells, treated with or without Cis for 24 hours. **f** q-PCR analysis of *Xbp1s* and *Xbp1u* in KPC cells treated with Cis for various hours (n = 6, combined from two independent biological repeats). **g** q-PCR analysis of *Xbp1s* in KPC cells treated with Cis (20 μM), 5FU (20 μM), Rsl3 (20 μM), or MRTX1133 (20 μM) for 22 hours (n = 4, representative of two independent biological repeats). The relative intensity of proteins (normalized to GAPDH or β-tubulin) is shown below the blots (**c**–**e**). All values are presented as means ± SEM. Statistical significance was determined using an unpaired, two-tailed Student’s t-test (**f**, **g**). *, p < 0.05; **, p < 0.01; ***, p < 0.001; n.s. not significant.

We hypothesized that if there are any negative regulator(s) of STING signaling intrinsic to PDAC, the cells may downregulate such factor(s) to enhance pro-survival STING signaling during chemotherapy adaptation. To test this, we examined the suppressed genetic signatures and pathways in PDAC cells under chemotherapy. Transcriptomic analysis revealed inhibition of the protein secretion pathway during cisplatin treatment, suggesting potential disruptions in ER protein homeostasis pathways, as secreted proteins are initially synthesized and processed in the ER (Fig. 3a, b). We therefore examined changes in key regulators of ER protein homeostasis, which are mainly composed of the unfolded protein response (UPR) and ER-associated degradation (ERAD) pathways and have been implicated in the regulation of STING [8, 39, 40]. Upon prolonged exposure to cisplatin, the protein level of IRE1α in the UPR pathway consistently decreased (Fig. S2a), while levels of BIP, PERK, and key proteins in the ERAD pathway, HRD1 and SEL1L, remained largely unchanged (Fig. 3c). Phos-tag gel analysis confirmed that the reduction in IRE1α band was not due to a band upshift or activation, as no phosphorylated IRE1α was detected (Fig. S2b). Consistent with these findings, reduced IRE1α protein levels were observed across tumor of PDAC patients post-chemotherapy (Fig. 3d). The decrease in IRE1α protein levels appears to be a general response to chemotherapy, as similar reductions were observed in PDAC cells from patient-derived xenograft (PDX) mice treated with cisplatin and in other cancer cell lines, such as human pancreatic cancer MIA PaCa-2 cells, mouse breast cancer 4T1, and hepatoma Hepa1-6 (Fig. 3e). This effect was consistent across various chemotherapeutic agents, including 5-fluorouracil and oxaliplatin (Fig. S2c).

A primary function of IRE1α is to splice X-box binding protein 1 (XBP1) via its RNase domain, generating the transcription factor XBP1s, which plays a critical role in the ER stress response [28, 41, 42, 43]. We observed a significant reduction in *Xbp1s* mRNA levels following cisplatin and 5-fluorouracil treatment, corroborating the decrease in IRE1α function (Fig. 3f, g). To determine if this effect was specific to chemotherapy, we assessed *Xbp1s* mRNA levels after treatment with the ferroptosis inducer Rsl3 and the KRAS inhibitor MRTX1133, finding no significant reduction (Fig. 3g). Electron microscopy revealed no notable changes in ER or mitochondria structure after 24 hours of cisplatin treatment (Fig. S2d). Additionally, no significant changes were observed in mitochondrial proteins, including the dynamics regulators Dynamin-related protein 1 (DRP1) and Mitofusin 2 (MFN2), the transporting protein Voltage-dependent anion channel (VDAC), the chaperone Heat shock protein 60 (HSP60), and the respiratory chain protein complexes (Fig. S2e). Furthermore, autophagy induction, as indicated by LC3B, did not show any significant alterations (Fig. S2e). Thus, the substantial decrease in IRE1α expression is likely a regulated process rather than a consequence of organelle damage or cellular injury induced by cisplatin. In summary, our results indicate that chemotherapy specifically suppress protein levels of IRE1α, a reported regulator of the STING signaling [40].

### IRE1α negatively regulates STING and STING-mediated inflammation

**Fig. 4:**
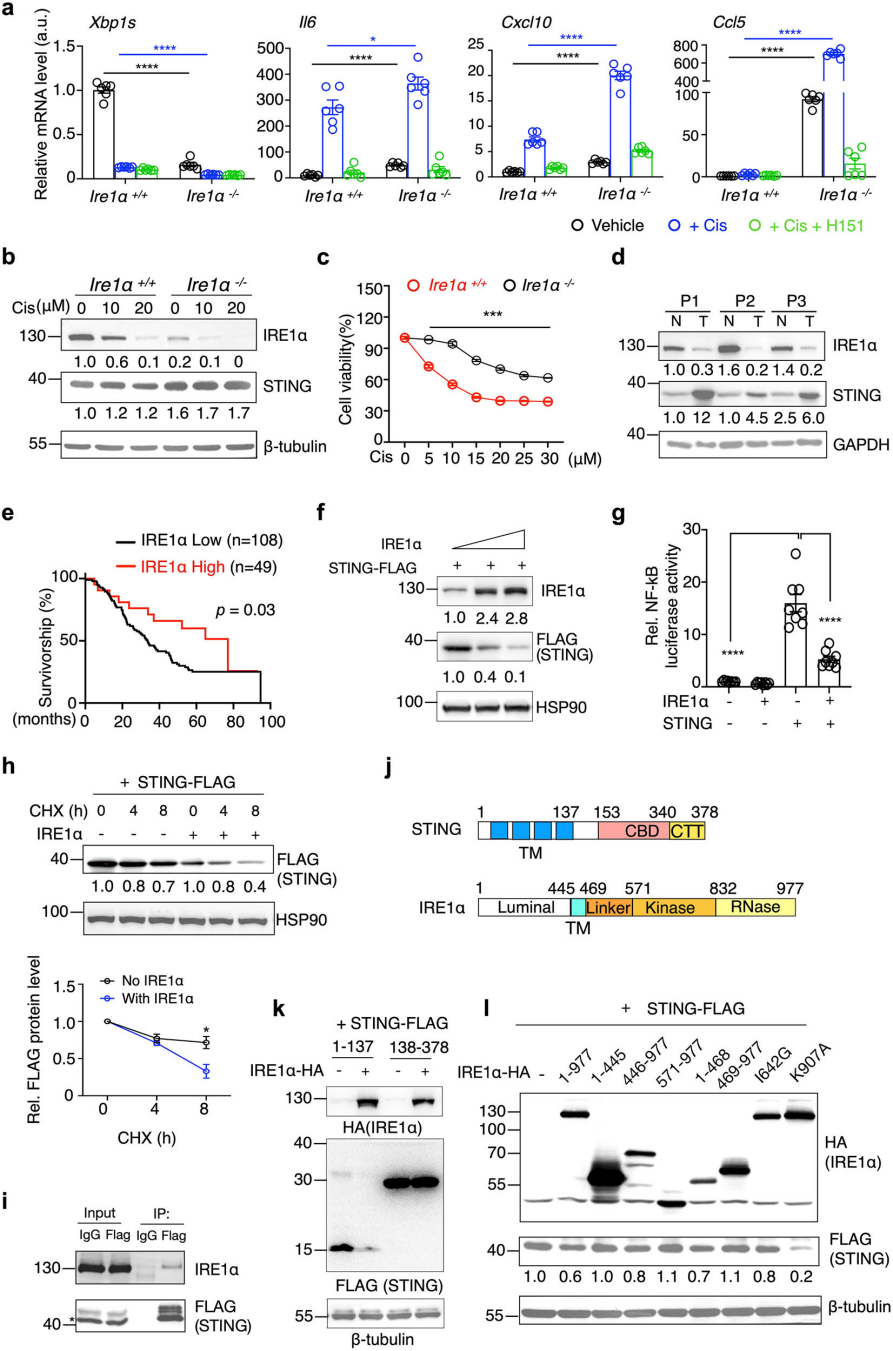
IRE1α directly regulates STING and its signaling in PDAC. **a** q-PCR analysis of indicated genes in *WT* and *Ire1α*^-/-^ KPC cells treated with vehicle, Cis, or in combination with H151(4 μg/mL, pretreated 1 hour prior to Cis treatment) (n = 6, combined from two independent biological repeats). **b** Immunoblot analysis in *WT* and *Ire1α*^-/-^ cells treated with various concentrations of Cis, representative of three independent biological repeats. **c** CCK-8-based cell viability assay of *WT* and *Ire1α*^-/-^ KPC cells treated with different doses of Cis for 24 hours (n = 5, representative of two independent biological repeats). **d** Immunoblot analysis of tumor (T) and adjacent non-tumor (N) tissues from PDAC patients. **e** Kaplan–Meier survival analysis for IRE1α expression (high vs. low) in pan-cancer from the ICGC/TCGA cohort via cBioPortal database. **f** Immunoblot analysis in HEK293T cells transfected with STING and IRE1α plasmids, representative of three independent biological repeats. **g** NF-κB dual-luciferase assay in HEK293T cells transfected with STING and IRE1α plasmids (n = 8, combined from two independent biological repeats). **h** Translation shut-off assay in HEK293T cells transfected with STING ± IRE1α plasmid, treated with cycloheximide (CHX, 50 μg/mL) for indicated times; quantitation from 3 independent repeats shown below. **i** Immunoblot analysis following immunoprecipitation of exogenous STING-FLAG from HEK293T cells transfected with STING and IRE1α plasmids. IgG immunoglobulin G, IP immunoprecipitation. * indicates the target band. Results are representative of three independent biological repeats. **j** Diagrams of the STING and IRE1α protein domains. STING: TM transmembrane (amino acids 1-137), CBD c-di-GMP-binding domain (amino acids 153-340), CTT carboxy-terminal tail (amino acids 340-378). IRE1α: Luminal, luminal domain (amino acids 1-445); TM, transmembrane (amino acids 445-469); Linker (amino acids 469-571); Kinase (amino acids 571-832); RNase (amino acids 832-977). **k** Immunoblot analysis of STING-FLAG protein levels in HEK293T cells transfected with truncated STING and full-length of IRE1α plasmids, representative of two independent biological repeats. **l** Immunoblot analysis of STING-FLAG protein levels in HEK293T cells transfected with full-length/ truncated IRE1α plasmids, IRE1α mutants (I642G, kinase loss-of-function mutation; K907A, RNase loss-of-function mutation), and full-length STING plasmids, representative of three independent biological repeats. The relative intensity of proteins (normalized to GAPDH, β-tubulin or HSP90) is shown below the blots. All values are presented as means ± SEM. Statistical significance was determined using unpaired, two-tailed Student’s t-test (**a**, **c**, **g**, **h**) or log-rank test (**e**). *, p < 0.05; ***, p < 0.001; ****, p < 0.0001.

To investigate the role of IRE1α in chemotherapy response in pancreatic cancer, we generated IRE1α knockout (KO) pancreatic cancer cell lines. IRE1α KO cells exhibited increased expression of inflammatory genes at baseline and following chemotherapy, which was suppressed by the STING inhibitor H151, suggesting that IRE1α negatively regulates STING-mediated inflammation in PDAC during chemotherapy (Fig. 4a). Toll-interacting protein (TOLLIP) has been implicated in stabilizing STING, with its absence leading to STING degradation in an IRE1α-dependent manner [40]. To elucidate whether IRE1α regulates STING protein stability in PDAC, we measured STING protein levels in IRE1α KO cells, observing an elevation compared to control cells (Figs. 4b and S3a). Consistent with the protective role of STING signaling in PDAC resistance to chemotherapy (Fig. 2a–d), IRE1α KO cells exhibited increased tolerance to chemotherapy in vitro (Fig. 4c). Notably, IRE1α protein levels were reduced, while STING protein levels were elevated in tumors from PDAC patients compared to adjacent non-tumor tissues (Fig. 4d). Analysis of the cBioPortal database also revealed a positive correlation between IRE1α expression and patient survival (Fig. 4e), underscoring the pathophysiological significance of IRE1α protein levels in tumors in vivo.

Mechanism of IRE1α-mediated STING inhibition was further clarified, and studies in HEK293T cells demonstrated that co-transfection with an IRE1α plasmid inhibited STING protein levels and downstream response while leaving cGAS protein levels unaffected (Fig. S3b, c). A dose-dependent inhibition of STING by IRE1α was further confirmed (Fig. 4f), and cisplatin treatment accelerated IRE1α-mediated STING degradation (Fig. S3d). Luciferase assays confirmed that IRE1α suppresses NF-κB activity downstream of STING (Fig. 4g). We then performed a translation shut-off assay using cycloheximide (CHX), which confirmed that IRE1α accelerates STING degradation (Fig. 4h). Using the proteasome inhibitor MG132 and lysosome inhibitor chloroquine (CHL), we found that MG132 inhibited IRE1α-induced STING degradation, whereas CHL had mild effect, suggesting a proteasome-dependent degradation pathway (Fig. S3e).

Immunoprecipitation (IP) assays demonstrated a direct interaction between IRE1α and STING (Fig. 4i), while no interaction was detected between IRE1α and cGAS, or between STING and other ER stress-related proteins (Fig. S4a, b). Both STING and IRE1α are transmembrane proteins (Fig. 4j). Co-expression of IRE1α with truncated STING constructs revealed that the transmembrane N-terminal region of STING is both necessary and sufficient for IRE1α-mediated degradation, while the C-terminal cytosolic region of STING is not involved (Fig. 4k). Additionally, the transmembrane region of IRE1α itself (amino acids 446-468) is essential for STING degradation by either N-terminal (amino acids 1-468) or C-terminal (amino acids 446-977) truncations of IRE1α (Fig. 4l, lanes 3 vs 6, lanes 4 vs 7). Notably, with loss-of-function mutations in the RNase (K907A) or kinase (I642G) domains of IRE1α, STING reduction was still observed, suggesting that this regulatory process is independent of IRE1α's RNase and kinase activities (Fig. 4l, lanes 8 and 9). Furthermore, overexpression of XBP1s in the IRE1α KO KPC cells did not mitigate the increased inflammation caused by cisplatin treatment (Fig. S4c), suggesting that IRE1α's regulation of STING and inflammation operates independently of XBP1s. Taken together, these data suggest that IRE1α directly interacts with STING and promotes STING degradation via their transmembrane domains, thereby regulating STING signaling in pancreatic cancer cells.

### Cisplatin downregulates *Ire1α* mRNA translation in PDAC

**Fig. 5:**
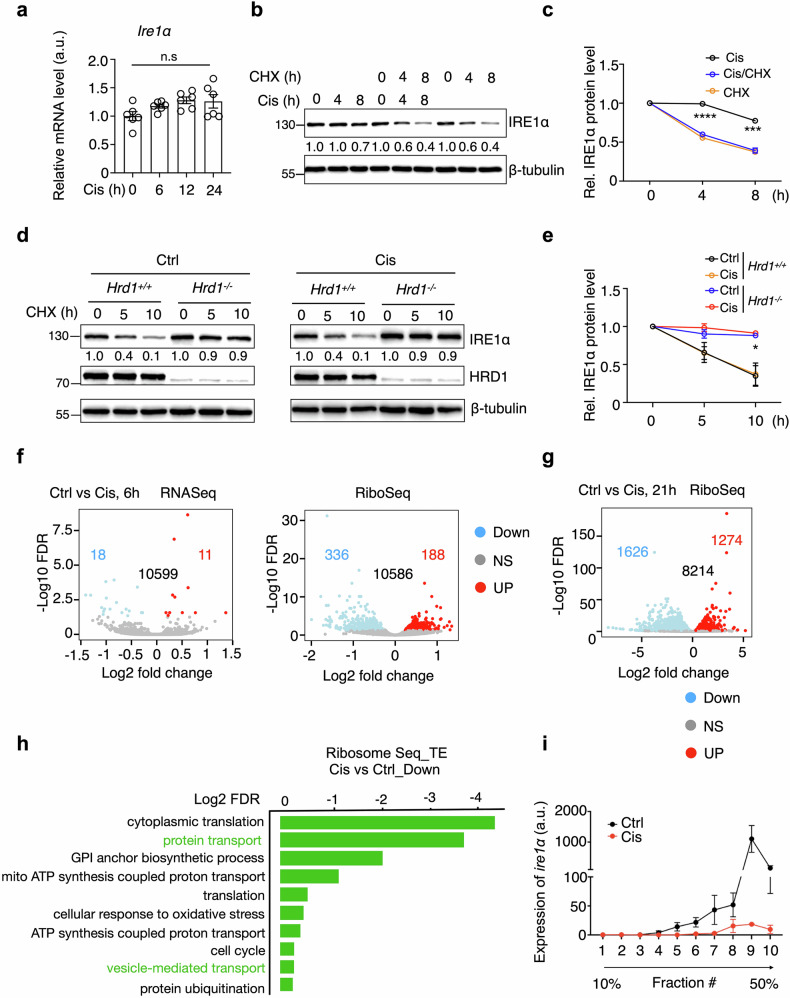
Chemotherapy inhibits IRE1α translation. **a** q-PCR analysis of *Ire1α* in KPC cells treated with 20 μΜ Cis for indicated times (n = 6, combined from 2 independent repeats). Immunoblot analysis in KPC cells treated with Cis ± CHX for indicated durations (**b**), with quantitation from 3 independent repeats shown in (**c**). Immunoblot analysis of *WT* and *Hrd1*^*-/-*^ MEF cells treated with vehicle control or 10μΜ Cis, followed by CHX for indicated durations (**d**), with quantitation from 3 independent repeats shown in (**e**). RNA-Seq and ribosome profiling of KPC cells treated with Cis for 6 hours (**f**) or 21 hours (**g**). Downregulated genes are shown in blue, upregulated in red (n = 2 for each time point). **h** Ribosome sequencing and translation efficiency (TE) analysis after 21 hours of Cis treatment. TE calculated as ribo/RNA. **i** Ribosome fractionation followed by q-PCR analysis of *Ire1α* mRNA in KPC cells treated with Cis for 21 hours (n = 3 control, n = 2 Cis, combined from 2 independent repeats). All values are presented as means ± SEM. Statistical significance was determined using unpaired, two-tailed Student’s t-test (**a**, **c**, **e**, **i**). *, p < 0.05; ***, p < 0.001; ****, p < 0.0001; n.s. not significant.

We aimed to uncover the mechanisms responsible for the reduction of IRE1α protein levels following chemotherapy. Initially, we measured *Ire1α* mRNA transcription levels and found that the reduction in IRE1α protein occurs at the post-transcriptional level, as no decreases in mRNA levels were detected after chemotherapy treatment (Fig. 5a). To elucidate whether cisplatin influences IRE1α protein stability, we performed a cycloheximide (CHX) chase assay to monitor protein degradation. Our results indicated that cisplatin treatment did not accelerate IRE1α protein degradation (Fig. 5b, c). IRE1α has been identified as a substrate for the E3 ubiquitin ligase HRD1, which targets it for proteasomal degradation [43]. Consistent with this, IRE1α protein was stabilized in HRD1 knockout (KO) MEF cells and KPC cells (Figs. 5d, e and S4d). Western blot analysis in HRD1 KO cells revealed no increase in IRE1α degradation following cisplatin treatment (Figs. 5d, e and S4d), indicating that the reduction of IRE1α protein post-chemotherapy likely involves a degradation-independent mechanism.

We then determine whether chemotherapy affects IRE1α levels by inhibiting its translation.

Ribosome sequencing revealed that cisplatin treatment for 6 hours induced a prompt shift in the cellular translation profile, with 188 mRNAs upregulated and 336 mRNAs downregulated (Figs. 5f and S5a–e). In contrast, transcriptional analysis showed only a modest change, with 18 genes downregulated and 11 genes upregulated (Fig. 5f). These findings highlight a rapid, translation-dependent regulation of protein levels in response to chemotherapy. Cisplatin treatment for 21 hours led to the upregulation of translation for 1274 mRNAs and downregulation of translation for 1626 mRNAs (Figs. 5g and S5a–e), with a notable impact on genes involved in protein transport and vesicle-mediated transport (Fig. 5h). The inhibition of protein synthesis during Cis treatment was further validated using a puromycin incorporation assay (Fig. S6a, b). In addition, polysome profiling specifically showed a reduction in the association of *Ire1α* mRNA with polysomes after cisplatin treatment, indicating inhibited translation of *Ire1α* (Fig. 5i). In summary, these data suggest that chemotherapy reduces IRE1α protein levels by inhibiting its translation.

### Chemotherapy increases cancer cell susceptibility to ER stress-induced cell death

**Fig. 6:**
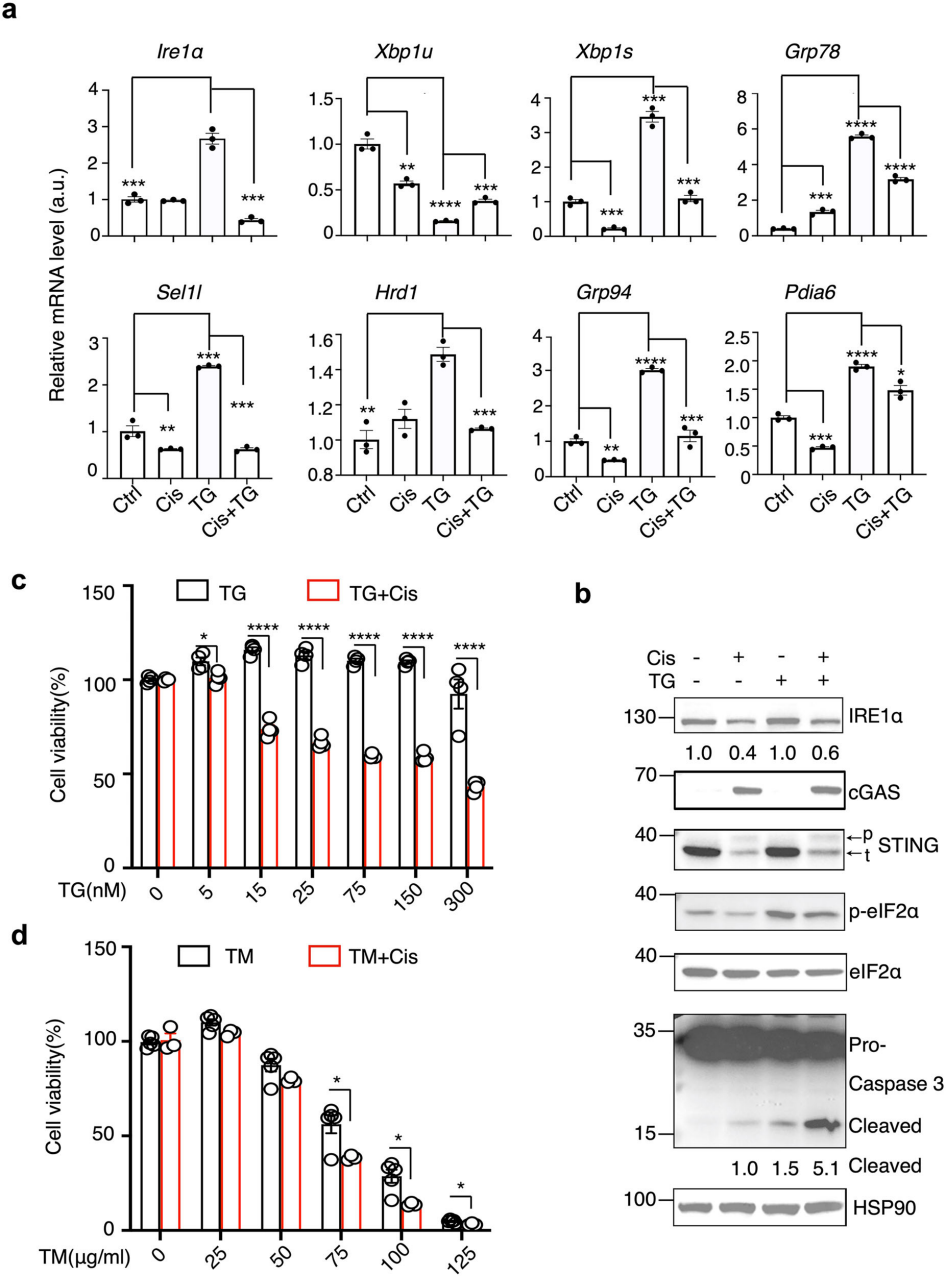
Chemotherapy sensitizes cancer cells to ER stress inducers. **a** q-PCR analysis of indicated genes in KPC cells treated with 20 μΜ Cis and Thapsigargin (TG,100 nM) for 24 hours (n = 3, representative of two independent biological repeats). **b** Immunoblot analysis of KPC cells treated with Cis ± TG for 24 hours, Blots are representative of two independent biological repeats. Cell viability analysis of KPC cells treated with Cis and varying concentrations of TG (**c**) or Tunicamycin (TM, **d**) for 24 hours (n = 3-5, combined from 2 independent repeats). Data are presented as mean ± SEM. Statistical significance was evaluated using an unpaired, two-tailed Student’s t-test (**a**, **c**, **d**). *, p < 0.05; **, p < 0.01; ***, p < 0.001; ****, p < 0.0001.

Given the pivotal role of IRE1α-XBP1s signaling pathway in enhancing protein folding capacity to manage ER stress, we investigated the impact of chemotherapy on this response. Thapsigargin (TG), a known ER stress inducer, markedly upregulated the level of *Xbp1s* and its target genes, including chaperones such as *Grp78* and *Grp94*, as well as ERAD components *Sel1l*, *Hrd1*, and the protein disulfide isomerase *Pdia6* in PDAC cells (Fig. 6a). In contrast, cisplatin treatment mitigated the IRE1α-mediated UPR responses (Fig. 6a). This effect was consistently observed with tunicamycin (TM), another ER stress inducer (Fig. S7a, b). Combined treatment with cisplatin and TG did not alter PERK signaling, as indicated by eIF2α phosphorylation; however, cleaved caspase-3 was synergistically increased following the combined treatment (Fig. 6b, lanes 4 vs. 3). In vitro cell viability assays further confirmed that this combined treatment increased cell death compared to cisplatin alone, with the addition of either TG (Fig. 6c) or TM (Fig. 6d) at indicated dosages. Based on these findings, we might devise a novel therapeutic strategy that combines chemotherapy with ER stress inducers.

### Exacerbating proteostasis imbalance enhances the efficacy of chemotherapy

We demonstrated that abolishing STING signaling in PDAC cells (*Sting*^*-/-*^) sensitizes cancer cells to chemotherapy (Fig. 2c, d). Encouraged by in vitro results of combining chemotherapy with ER stress inducer (Fig. 6c, d), we evaluated whether this combined treatment could further enhance therapeutic efficacy in vivo, building on the impact of STING signaling inhibition.

**Fig. 7:**
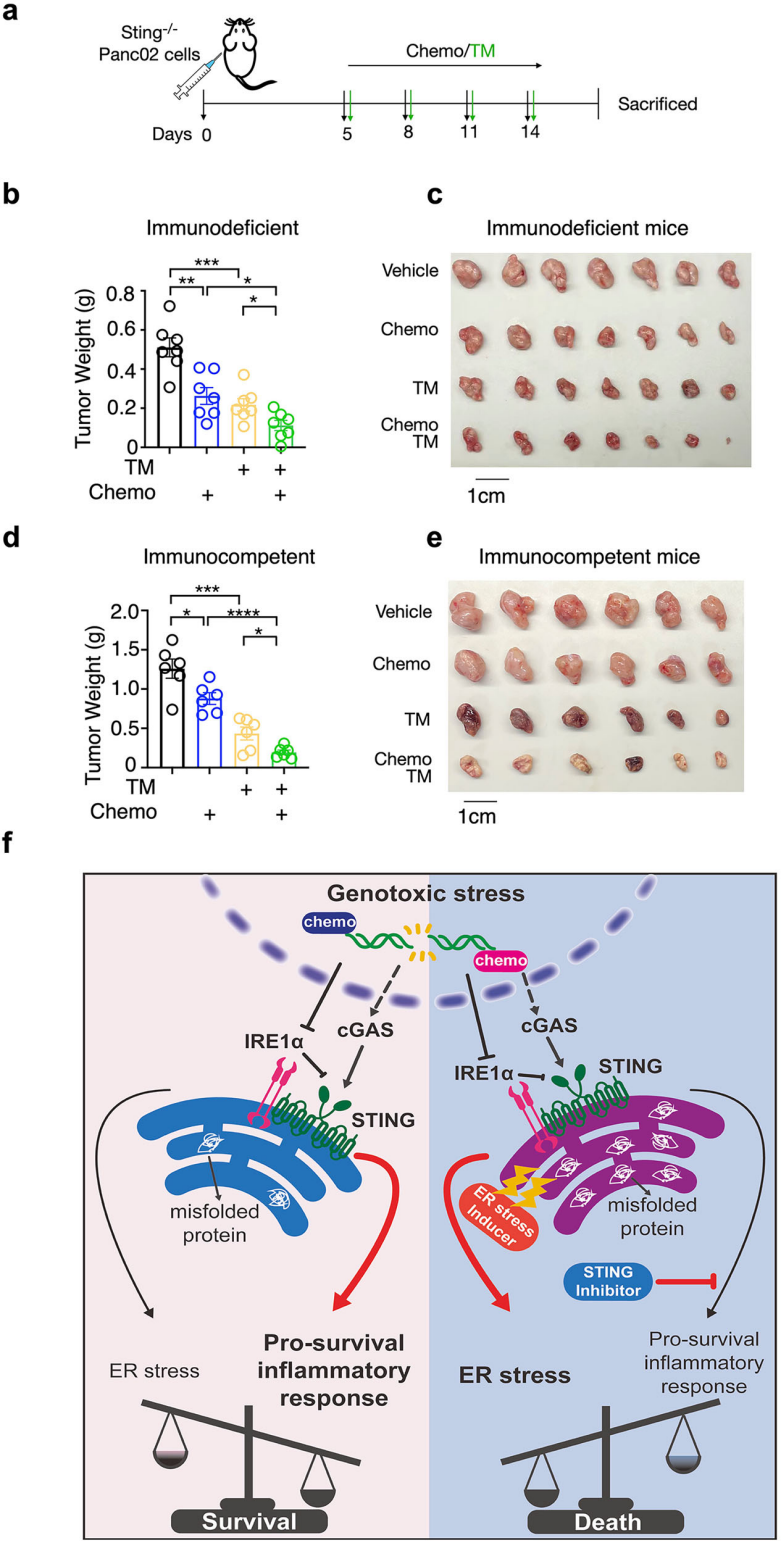
Targeting STING and ER homeostasis enhances the efficacy of chemotherapy. **a** Schematic of cancer model: *Sting*^*-/-*^ Panc02 cell -transplanted mice received four intraperitoneal injections of Chemo drugs (Cis, 3 mg kg^−1^; 5FU, 25 mg kg^−1^; Irinotecan, 25 mg kg^−1^) and four peritumoral TM injections (TM, 0.3 mg kg^−1^). Tumors from immunodeficient (**b**, **c**) or immunocompetent (**d**, **e**) mice at the end of experiment (Day 16 and Day 19, respectively). Tumors weight quantification in immunodeficient mice (**b**, n = 7 mice per group), and in immunocompetent mice (**d**, n = 6 mice per group), representative of two independent repeats. **f** Proposed model illustrating the synchronized effect of STING pathway inhibition and ER stress induction, in conjunction with chemotherapy. STING is negatively regulated by IRE1α in PDAC, while chemotherapy-induced downregulation of IRE1α amplifies STING signaling and promotes cancer cell survival. However, this dysregulated proteostasis network increases susceptibility to ER stress-inducing agents. All values are presented as mean ± SEM. Statistical significance was determined using an unpaired, two-tailed Student’s t-test (**b**, **d**). *, p < 0.05; **, p < 0.01; ***, p < 0.001; ****p < 0.0001.

Immunodeficient mice were subcutaneously transplanted with STING KO Panc02 cells and treated with chemotherapy, TM, or both. The combination therapy significantly enhanced the antitumor efficacy of chemotherapy, as demonstrated by a substantial reduction in tumor size in the combination-treated mice compared to chemotherapy alone (Fig. 7a–c), indicating an intrinsic effect on cancer cells. This enhanced efficacy of combined treatment was also observed in immunocompetent mice (Fig. 7d, e), ruling out potential negative impact of this strategy on the host immune system's anti-tumor response. Furtherly, to better mimic the human clinical setting where STING is expressed, we conducted in vivo experiments comparing tumor growth in mice transplanted with WT PDAC cells and treated with chemotherapy alone, chemotherapy combined with TM, or chemotherapy combined with both TM and STING antagonist H151. Notably, the combination of TM and H151 resulted in the greatest suppression of tumor growth under chemotherapy (Fig. S7c). Therefore, we proposed a model illustrating the synchronized effect of STING pathway inhibition and ER stress induction in conjunction with chemotherapy (Fig. 7f), highlighting the potential of this approach to enhance chemotherapy efficiency and serve as a promising therapeutic strategy.

## Discussion

PDAC is an exceptionally aggressive malignancy, often diagnosed at an advanced stage when curative surgical resection is no longer an option. Chemotherapy remains a standard treatment across various stages of PDAC; however, its response rate is exceedingly low [4]. Therefore, analyzing the intracellular signaling network reprogramming that occurs after chemotherapy and developing targeted strategies to improve PDAC chemotherapy efficacy are essential. Our study delineated the altered signaling landscape, its underlying molecular mechanisms, and its pathophysiological significance in chemotherapy-treated PDAC. We further proposed a combinatorial treatment strategy integrating chemotherapy, STING inhibition, and ER stress induction and achieved optimal therapeutic efficacy (Fig. 7f).

STING, a critical mediator of the cellular response to cytosolic DNA, plays a pivotal role in linking DNA damage and inflammation [16, 22]. With genetic deletion or chemical inhibition of STING, our data showed cisplatin and 5FU induced STING-mediated inflammation in PDAC, mounting a pro-tumor survival effect both in vitro and in immune-deficient or immune-competent animals (Fig. 2a–d). Canonically, STING agonists induce the exit of STING from the ER and its trafficking to the TGN for activation and recruitment of downstream effectors in innate immune cells [8], and activated STING is subsequently trafficked to endolysosomes for degradation [13, 14]. Our studies showed that a fraction of STING flows out of the ER for phosphorylation and activation upon cisplatin or DMXAA treatment in PDAC cells (Fig. 2e, f). However, unlike in macrophages, this activated STING fraction in PDAC does not appear to undergo lysosomal degradation (Fig. 2g, h), highlighting the unique nature of STING trafficking and signaling regulation in cancer cells. Future research should focus on delineating the precise molecular mechanisms by which STING is modulated in the ER and post-ER organelles of cancer cells under chemotherapy, which might facilitate the development of other therapeutic strategies to suppress tumor-intrinsic pro-survival STING signaling for cancer treatment.

IRE1α, the most ancient and conserved ER-resident sensor of misfolded proteins, activates its ribonuclease activity upon activation to splice *Xbp1* mRNA, producing the transcription factor XBP1s, which expands ER volume and enhances protein-folding capacity [28, 41, 42]. By restoring proteostasis, IRE1α plays a critical role in the resistance of pancreatic cancer to KRAS inhibitor therapies [44]. More recently, IRE1α has been shown to participates in broad biological and pathological processes, including pain, muscle regeneration, thermogenesis, and pyroptosis, which are independent of the regulation of ER protein homeostasis but related to IRE1α's RNase activity to degrade target mRNAs in a context-dependent manner [45–48]. IRE1α can mediate the degradation of ER-resident STING via lysosomal degradation in Toll-interacting protein (TOLLIP)-deficient mouse embryonic fibroblasts [40], while being dispensable for the negative regulation of ER-resident STING levels in macrophages under homeostatic conditions [39]. In this study, we found that IRE1α serves as a negative modulator of chemotherapy-induced STING signaling in PDAC by promoting STING protein degradation through their transmembrane domains, independently of IRE1α's RNase activity or XBP1s (Fig. 4k, l). Particularly, IRE1α mRNA translation and protein levels in PDAC were significantly downregulated following chemotherapy, enabling an enhanced STING-mediated pro-survival response. Taken together, beyond its role as a key signaling hub for maintaining ER homeostasis, the IRE1α pathway functions as a master regulator of various critical signaling pathways, including the regulation of STING signaling in PDAC, which can be reprogrammed by PDAC cells to adapt to chemotherapy.

We found that chemotherapy drugs, in a STING dependent manner, cause PDAC cells to up-regulate multiple inflammatory cytokines, which have the effect of promoting tumor survival and antagonizing cellular immunity in a variety of tumors [49, 50]. Among the diverse repertoire of inflammatory cytokines upregulated, *Il6* transcription in KPC cells following chemotherapy increased by over 200-fold (Fig. 1c, g, h), accompanied by a substantial elevation in IL-6 secretion (Fig. 1d). While tumor cell-intrinsic STING signaling has been reported to promote tumor cell survival in an IL-6–dependent manner [16, 31], the precise contribution of IL-6 to this effect in PDAC, as well as the potential roles of other STING-driven processes such as NF-κB activation and autophagy induction, warrants further study. Of note, our results show that cisplatin induces an altered translational program in PDAC cells, leading to downregulation of translation for 1626 mRNAs at the 21 hours time point (Fig. 5f, g). The top genes with decreased translation are enriched in protein transport and vesicle-mediated transport pathways, but not in inflammatory response pathways (Fig. 5h). Previous studies have reported that STING activation can reprogram cellular mRNA translation, promoting an inflammatory- and survival-favored translational program in a PERK-eIF2α-dependent manner [51]. Additionally, while nutrient deprivation leads to a substantial global inhibition of protein translation, selective increased translation of cytokine and inflammatory mRNAs has been observed in starved cancer cells, also dependent on eIF2α signaling [52]. Of note, PERK protein levels (Fig. 3c) and eIF2α phosphorylation (Fig. 6b) remain largely intact in PDACs following chemotherapy. Whether the maintenance of inflammatory gene translation amid substantial translational arrest in chemo-treated PDAC cells involves the PERK-eIF2α axis or the broader integrated stress response warrants future investigation.

Our studies show tumor cells selectively downregulate the IRE1α pathway to amplify the production of the pro-survival inflammatory molecules, but this rewired proteostasis network renders cancer cells highly vulnerable to ER stressors. Furthermore, combining chemotherapy with the inhibition of cancer cell-intrinsic STING and disruption of the ER proteostasis network can significantly enhance the effectiveness of PDAC treatment. Therefore, the intricate relationship between chemotherapy, proteostasis, IRE1α, and STING underscores the complexity of cellular stress responses in cancer. Understanding these interactions opens new avenues to precisely target vulnerabilities of cancer cells. Future research should focus on developing targeted drug delivery systems capable of precisely delivering cisplatin, ER stress inducers, and STING inhibitors directly to cancer cells, thereby enhancing the efficacy and minimizing the toxicity of this combined treatment strategy. Additionally, investigating whether these combination strategies can improve chemotherapy sensitivity in other gastrointestinal tumors, particularly those characterized by prominent proteostasis and inflammatory signatures, represents a promising avenue for future exploration.

## Materials and methods

### Ethics approval

Human PDAC tissue specimens were obtained from the Department of Hepatobiliary and Pancreatic Surgery, the First Affiliated Hospital, Zhejiang University. The human research protocols were approved by the Clinical Research Ethics Committee of the First Affiliated Hospital, Zhejiang University School of Medicine (Approval No. IIT20230213B). Written informed consent was obtained from each patient at the time of enrollment.

### Mice

Male athymic Nude and C57BL/6 mice (6–8 weeks old) were obtained from Shanghai SLAC

Laboratory Animal Co. and housed under controlled conditions (20°C, 40-60% humidity) with a 12-hour light/dark cycle. They were fed a standard chow diet (LabDiet 5LOD:13% fat, 57% carbohydrate, 30% protein). All experimental procedures involving animals were approved by the Animal Experimentation Ethics Committee of the First Affiliated Hospital, School of Medicine, Zhejiang University, in compliance with institutional and national guidelines for animal welfare.

### In vivo tumor studies

The PDAC KPC cell line, derived from *Kras*^*LSL-G12D*^*; Trp53*^*LSL-R172H*^*; Pdx1-Cre* mouse tumors, was provided by Dr. R. Kalluri (MD Anderson Cancer Center), while Panc02 cell line was obtained from ATCC (USA). For tumor implantation, 7.0 ×10^5^ KPC cells or 1.0 ×10^5^ Panc02 cells were injected into the right flank of mice. Five to seven days post-transplantation, mice were randomized into groups for drug treatment. Drug administration was performed as described. The chemotherapy group received intraperitoneal injections of cisplatin (Cis, 3 mg kg^−1^), 5-fluorouracil (5FU, 25 mg kg^−1^), and irinotecan (25 mg kg^−1^). H151 (5 mg kg^-1^) was administered intraperitoneally, and Tunicamycin (TM,0.3 mg kg^−1^) peritumorally. All drugs were obtained from MedChemExpress (MCE). Tumor growth was monitored, and tumors were harvested when remained <10% of body weight and dimension <20 mm, per ethical guidelines.

### Survival curve analysis of cancer patients

Survival curves were generated based on IRE1α expression levels using data from the cBioPortal database. Median IRE1α expression was calculated based on mRNA z-scores relative to all samples (log FPKM values). Patients with hazard ratios ranging from -2.51 to -0.06 were classified as IRE1α Low (n = 108), while those with hazard ratios from -0.06 to 4.83 were classified as IRE1α High (n = 49).

### Cell culture and drug treatment

HEK293T, MEF, Raw264.7 cells were obtained from ATCC and cultured in DMEM medium (Cytiva), supplemented with 10% FBS (Braserum) and 1% penicillin/streptomycin at 37°C under 5% CO_2_. Drug treatments (all from MCE) were administered at optimized concentrations: Cis (20 μM), 5FU (20 μM), oxaliplatin (20 μM), DMXAA (20 μg/mL), H151 (4 μg/mL, 1-hour pretreatment), thapsigargin (TG,100 nM), tunicamycin (TM, 5 μg/mL), cycloheximide (CHX,50 μg/mL), MG132 (25 μM), brefeldin A (BFA, 5 μg/mL), Bafilomycin A1(BafA1, 20 nM), chloroquine (100 μM), Rsl3 (20 μM), MRTX1133 (20 μM), ATM inhibitor KU-55933 (1 μM), and ATR inhibitor VE-821 (1 μM). All treatments were conducted under sterile conditions with optimized incubation times for each setup.

### CRISPR-mediated gene KO cells

CRISPR-Cas9-mediated knockout (KO) of IRE1α and STING was achieved using the lentiCRISPRv2 vector, developed by the Zhang laboratory at MIT, following previously described protocols [53]. The lentiCRISPRv2 vector was engineered to express the single guide RNA (sgRNA), Cas9 protein, and a puromycin resistance gene for selection. The sgRNAs targeting *Ire1α* and *Sting* were designed, synthesized, and cloned into the vector. The specific sgRNA sequence for *Sting* was: 5’-TGAGGGCTACATATTTGGAG-3’. The specific sgRNA sequence for *Ire1α* was: 5’-CAGGGTCGAGACAAACAACA-3’. Successfully transduced cells were selected using puromycin to establish stable knockout cell lines for subsequent experiments. Sanger sequencing of the targeted loci was further performed to confirm indel formation at the expected sites in knockout (KO) cells used in the experiments.

### Western blot and image quantitation

For protein isolation from cells, cell lysates were prepared as described in previous studies *(39,53)*. Briefly, cells were lysed in RIPA Lysis Buffer (Beyotime), supplemented with phosphatase and protease inhibitors (MedChemExpress). After centrifugation at 20,000 g at 4°C for 10 minutes, the supernatant was collected, and protein concentration was determined using the BCA assay. For protein isolation from PDAC patient tumor samples, tissue specimens measuring approximately 5 mm × 5 mm × 5 mm were collected, finely minced using scissors, and homogenized. Protein isolation from these tumor samples was performed using the same method as described above for cell lysates. Samples were then separated using SDS-PAGE or Phos-Tag SDS-PAGE gel (APExBIO) and transfer to a polyvinylidene difluoride (PVDF) membrane (Millipore).

For Western blot analysis, the following antibodies were used: Flag (Sigma F1804; 1:2000), HA (Proteintech 51064-2-AP; 1:4000), HRD1 (Proteintech 13473-1-AP; 1:1000), IRE1α (Cell Signaling 3294; 1:3000), β-Tubulin (Proteintech 10068-1-AP; 1:3000), HSP90 (Proteintech 13171-1-AP; 1:5000), HSP60 (Proteintech 15282-1-AP; 1:5000), p-TBK1 (Ser172) (Cell Signaling 5483; 1:1000), TBK1 (Cell Signaling 38066; 1:2000), GAPDH (Abclonal AC033; 1:5000), STING (Proteintech 19851-1-AP; 1:2000), p-STING (Cell Signaling 72971; 1:1000), cGAS (Cell Signaling 31659; 1:2000), eIF2α (Cell Signaling 9722; 1:2000), p-eIF2α (Cell Signaling 3597; 1:1000), γ-H2AX (Abclonal AP0099; 1:2000), OXPHOS (Abcam ab110413; 1:5000), VDAC (Proteintech 10866-1-AP; 1:2000), LC3 (Proteintech 14600-1-AP; 1:2000), DRP1 (Proteintech 12957-1-AP; 1:2000), MFN2 (Proteintech 12186-1-AP; 1:2000), β-actin (Abclonal AC026;1:5000), p65 (Cell Signaling 8242; 1:2000), P-p65 (Cell Signaling 3033; 1:2000), GPX4 (Abclonal A1933; 1:1000), ERK1/2 (Cell Signaling 4696; 1:2000), p-ERK1/2 (Cell Signaling 4370; 1:2000), Caspase3 (Cell Signaling 14220; 1:1000), QRICH1 (Sigma-Aldrich HPA037677; 1:2000). Western blot band density was quantified using Image Lab Software version 4.1 on the ChemiDOC XRS+ system (Bio-Rad). Protein levels were normalized to loading controls such as HSP90, GAPDH, β-tubulin, or β-actin. Phosphorylated protein levels were normalized to their respective total protein levels as indicated as p/t. Data are expressed as the mean ± SEM unless otherwise noted. All full length uncropped original western blots used in the manuscript are uploaded in the Supplementary Materials file.

### Coimmunoprecipitation

The cells were lysed in a lysis buffer containing 150 mM NaCl, 1 mM EDTA, 50 mM Tris-HCl, a protease inhibitor cocktail (MedChemExpress), 0.5% Nonidet P-40 (NP-40), and 10 mM N-ethylmaleimide (NEM), on ice for 15 minutes. After lysis, the cell lysates were centrifuged at 20,000 g at 4°C for 10 minutes. The supernatants were collected, and protein concentration was measured using the Bradford assay. For immunoprecipitation, the respective antibody or control IgG was added to the supernatant and incubated overnight at 4°C. Following antibody incubation, 20 μL of Protein A/G PLUS-Agarose (Santa Cruz) was added to each sample and incubated for an additional 3 hours at 4°C. The agarose beads were then washed five times with the lysis buffer to remove nonspecific proteins. For elution, the immunocomplexes were boiled for 5 minutes in SDS sample buffer. The samples were then subjected to SDS-PAGE and analyzed by Western blot.

### Plasmid construction

Flag-tagged STING, cGAS, and HA-tagged IRE1α were cloned into the PCDH-CMV-MCS-EF1-Puro plasmid, a generous gift from Dr. Zhangsen Zhou at Shanghai Institute of Nutrition and Health, Chinese Academy of Science. All plasmids were constructed using standard molecular biology techniques and subsequently verified through DNA sequencing.

### RNA extraction, reverse transcription (RT) and quantitative PCR (Q-PCR)

Total RNA was isolated from cells using RNAiso plus kit (Takara). cDNA was generated with the PrimeScript™ RT reagent Kit, incorporating gDNA Eraser (Perfect Real Time, Takara), following the manufacturer's instructions. The cDNA was then amplified using TB Green Premix Ex Taq (Tli RNaseH Plus, Takara) according to the manufacturer's instructions. Q-PCR data were collected using the QuantStudio™ 5 (Applied Biosystems), with gene expression normalized to the ribosomal protein L32 gene for each sample.

The sequences of the primers used in this study are listed below:

*Il6* (AGACAAAGCCAGAGTCCTTCAG; TGCCGAGTAGATCTCAAAGTGA),

*Ccl5* (GCTGCTTTGCCTACCTCTCC; TCGAGTGACAAACACGACTGC),

*Ccl2* (TTAAAAACCTGGATCGGAACCAA; GCATTAGCTTCAGATTTACGGGT),

*Cxcl10* (AATCCGGAATCTAAGACCATCA; GCAATTAGGACTAGCCATCCAC)

*Xbp1s* (GAGTCCGCAGCAGGTG; GTGTCAGAGTCCATGGGA),

*Xbp1u* (ACTATGTGCACCTCTGCAGC; GTCCAGAATGCCCAAAAGG),

*Ire1α* (CTGTGGTCAAGATGGACTGG; GAAGCGGGAAGTGAAGTAGC),

*Hrd1* (AGCTACTTCAGTGAACCCCACT; CTCCTCTACAATGCCCACTGAC),

*Sel1l* (TGGGTTTTCTCTCTCTCCTCTG; CCTTTGTTCCGGTTACTTCTTG),

*Grp78* (TGTGGTACCCACCAAGAAGTC; TTCAGCTGTCACTCGGAGAAT),

*Grp94* (CTCAGAAGACGCAGAAGACTCA; AAAACTTCACATTCCCTCTCCA),

*Pdia6* (TGGTTCCTTTCCTACCATCACT; ACTTTCACTGCTGGAAAACTGC),

*Ifnb1* (AGATCAACCTCACCTACAGG; TCAGAAACACTGTCTGCTGG),

*L32* (GAGCAACAAGAAAACCAAGCA; TGCACACAAGCCATCTACTCA).

The Q-PCR conditions were: 94 **°**C for 5 min, 40 cycles of (94 **°**C for 15 sec, 58 **°**C for 15 sec and 72 **°**C for 30 sec), followed by dissociation curve analysis.

### Dual-luciferase reporter assay

Cells were transfected with the NF-κB luciferase reporter, TK Renilla, and the indicated plasmids using lipo8000 (Beyotime). Twenty-four hours post-transfection, whole-cell lysates were collected, and luciferase activity was measured using a dual-luciferase reporter assay system (Beyotime) according to the manufacturer’s instructions.

### Transwell assay

Transwell chambers (Corning, Inc.) without Matrigel were used for migration assays. A total of 500 µL of RPMI containing 10% FBS was added to the lower chambers, while 1×10^5^ cells suspended in serum-free RPMI were placed in the upper chambers. After 24 hours of culture, migrated cells were fixed with 4% paraformaldehyde and stained with crystal violet staining solution for 10 min at room temperature. Cells in the lower chamber were removed using a cotton-tipped swab. The picture was taken with light microscope (magnification, x100; Nikon Corporation).

### Cell Counting Kit-8(CCK-8) assay

To measure cell viability, cells were seeded in 96-well plates at a density of 1×10^5^ cells per well. The drugs were added to each well as the indicated concentration and incubated for 24 hours. Following treatment, 100 µL of complete culture medium containing 10 µL CCK-8 reagent (GlpBio) was added to each well. The plates were then incubated in the dark at 37°C for 1 hour. Absorbance was measured at 450 nm.

### Transmission electron microscopy (TEM)

KPC cells were seeded in six-well plates at a density of 1×10^6^ cells per well. The cells were then treated with either 20 µM Cisplatin or PBS as a control for 24 hours. Following treatment, the cells underwent fixation, staining, dehydration, and processing. Imaging was performed using a JEOL JEM-1400 transmission electron microscope, with services provided by the Electron Microscopy and Histology Core Facility at the Zhejiang University.

### Puromycin translation assay

KPC and Panc02 cells were seeded in 6-well plates and treated with cisplatin. Cells were then labeled with 10 μg/mL puromycin for 30 minutes. Subsequently, cells were washed twice with PBS, lysed in RIPA buffer containing protease inhibitors, and centrifuged to collect the supernatant. Newly synthesized proteins were detected by Western blot using anti-puromycin antibody (Abclonal, A23031; 1:2000).

### Ribosome sequencing

KPC cells were treated with 20 μM Cis or PBS for 6 or 21 hours in 6 cm dishes. After two PBS washes, cells were lysed with 500 μL buffer (8 mM HEPES pH 7.4, 80 mM KCl, 4 mM MgCl₂, 2% Triton X-100, 200 μg/mL CHX) and incubated on ice for 10 minutes. Lysates were centrifuged at 12,000 rpm for 12 minutes at 4°C, and supernatants were sent to Novogene for RNA-seq library preparation under RNase-free conditions. Separately, 900 ng of RNA was digested with RNase I (3 hours), extracted with TRIzol™ LS, denatured at 65°C, and resolved on a 15% TBE-Urea gel. RNA fragments (25-35 nt) were excised, eluted overnight at 4°C, and precipitated with isopropanol. Libraries were constructed using the NEBNext Small RNA Library Prep Set (E7330L) and sent to Novogene for sequencing.

### RNA-seq

Cells were washed, trypsinized, and resuspended in TRIzol Reagent for RNA extraction following the manufacturer's protocol. RNA-seq libraries were prepared using the VAHTS Universal V8 RNA-seq Library Prep Kit (Vazyme, NR605-02) and sequenced on the Illumina NovaSeq 6000 platform (150-bp paired-end). Quality control was performed with FastQC, and reads were aligned to the mouse genome (GRC38) using Salmon (v1.9.0). Differential gene expression analysis was conducted with DESeq2 (v1.38.3) in R (v4.2.1). Genes with adjusted p-value < 0.05 and log2 fold change > 1 were analyzed via Gene Set Enrichment Analysis (GSEA, v4.2.2). Heatmaps were created with the Complex Heatmap package (v2.14.0).

### Polysome profiling

KPC cells were treated with cisplatin or PBS for 21 hours, followed by treatment with 200 μg/mL cycloheximide for 10 min at 37 °C. The cells were then disaggregated with trypsin-EDTA (0.05%) for 10 min and washed twice with 200 μg/mL cycloheximide in 1× cold PBS. For polysome lysis, cells were resuspended in a lysis buffer composed of 15 mM Tris-HCl (pH 7.4), 15 mM MgCl_2_, 300 mM NaCl, 100 µg/mL cycloheximide, 1% Triton X-100, 40 U/µL RNase I, and 24 U/mL DNase. The mixture was incubated on ice for 10 minutes and then centrifuged at 12,000 × g for 10 minutes at 4 °C. Sucrose gradients were prepared using the BioComp Model 108 Gradient Master, employing 10% and 50% sucrose solutions (sucrose diluted in polysome buffer containing 15 mM Tris-HCl, 15 mM MgCl_2_, and 300 mM NaCl, all prepared under RNase-free conditions). The clear supernatants from lysed cells were loaded onto the 10% to 50% sucrose gradients and centrifuged at 150,000 × g (SW40 rotor, HIMAC CP80WX HITACHI) for 180 minutes at 4°C. Sucrose gradient fractions were collected using the ISCO gradient fractionation system (ISCO Model 160 Gradient Former Foxy Jr. Fraction Collector), and absorbance was monitored at 254 nm to record the polysome profile.

### Power analysis of the animal size

Based on sample size formula of the power analysis, N = 8(CV)^2^(1)/(PC)^2^, to reach the error = 0.05, Power = 0.80, percentage change in means (PC) = 20%, co-efficient of variation (CV) = 10 ~ 15% (varies between the experiments), 4-6 mice per group are the minimal number of mice to obtain statistical significance and to ensure adequate power.

### Statistical analysis

Results were presented as mean ± SEM, *p < 0.05; **p < 0.01; ***p < 0.001; ****p < 0.001. Group comparisons were performed using an unpaired two-tailed Student’s t-test. While normal distribution was assumed, it was not formally tested. No sample size calculations were conducted for in vitro experiments. All animals and samples were included without exclusions. Experiments were repeated at least twice or with independent samples.

## Supplementary information

Supplementary figure legends

Figure S1

Figure S2

Figure S3

Figure S4

Figure S5

Figure S6

Figure S7

Full length original western blots

## Data Availability

The RNA-seq data reported in this article has been deposited in National Genomics Data Center (*NGDC)* and are accessible through https://ngdc.cncb.ac.cn/gsa/s/A66t7je2. The Ribo-seq data reported in this article has been deposited in NCBI and accessible through https://www.ncbi.nlm.nih.gov/geo/query/acc.cgi?acc=GSE284365 (#GSE284365, secure token: uladcgqyhzojpqt). All data supporting the findings of this study are available from the corresponding author on reasonable request.
