# Supplementary material for: IRE1α translational suppression potentiates STING-dependent chemoresistance in pancreatic cancer
Source: Cell Death Dis. 2025 Oct 6;16(1):680. doi: 10.1038/s41419-025-07999-x (PMC12501023; doi:10.1038/s41419-025-07999-x)
Supplement: Supplementary file 1 — Supplementary figure legends [file 41419_2025_7999_MOESM1_ESM.docx]

**Supplementary materials**

**IRE1α Translational Suppression Potentiates STING-Dependent Chemoresistance in Pancreatic Cancer**

Yuan Luo^1, 5, 6^*, Mengqi Sun^1, 5^, Lei Chang^1, 5^, Zinan He ^2^, Xinghang Zhou^1^, Yaming Yuan^3^, Huijuan Sun^3^, Shiqi Luo^4^, Jinyan Huang^1^, Hongkun Wu^1^, Wenjun Liu^1^, Zhangsen Zhou^3^, Yuanhui Mao^4^, Yewei Ji^1, 2^*, Tingbo Liang^1^*

***Correspondence:** [liangtingbo@zju.edu.cn; ywji@cqmu.edu.cn](mailto:liangtingbo@zju.edu.cn;%20ywji@cqmu.edu.cn); luoyuan715@126.com

Include:

- 7 supplementary figures

**Supplementary figure legends**

**Figure S1. Chemotherapy induces inflammation via the STING pathway in PDAC.**
**a-b.** Immunoblot **(a)** and q-PCR **(b)** analysis in Panc02 cells treated with 20 μM Cis for 24 hours (n = 6-7, combined from 2 independent repeats). **c.** q-PCR analysis in *Sting^-/-^* Panc02 cells treated with 20 μM Cis for 24 hours (n = 3, representative two independent repeats). **d.** Quantification of tumor weights from immunodeficient mice that received four intraperitoneal injections of chemotherapy drugs (Cis 3 mg kg^−1^, 5FU 25 mg kg^−1^, Irinotecan 25 mg kg^−1^) alone or in combination with STING inhibitor H151 (5 mg kg^−1^). Tumor weights were measured at the end of the experiment (n = 6, 11, 7 mice, left to right, day 17, combined from two independent repeats). **e**. q-PCR analysis of inflammatory genes in KPC cells treated with Cis or DMXAA, ± BFA or BafA1 (n=4, representative of two independent biological repeats). **f.** q-PCR analysis in KPC cells treated with Cis, ATM inhibitor (KU-55933, 1μM) and ATR inhibitor (VE-821, 1μM), or their combination for 24 hours (n = 6, combined from two independent biological repeats). All values are presented as mean ± SEM. Statistical significance was determined using an unpaired, two-tailed Student’s t-test (**b-f**). *, p < 0.05; **, p < 0.01; ***, p < 0.001; ****, p < 0.0001.

**Figure S2. Chemotherapy drugs downregulate IRE1α protein levels in various cancer cell lines. a.** Quantification of IRE1**α** protein levels in KPC cells treated with 20 μM Cis for indicated hours (n=6). **b.** Immunoblot analysis of IRE1α protein levels and phosphorylation (phos-tag gel) in KPC cells treated with 20 μM Cis at various time points. KPC cells treated with Thapsigargin (TG, 100nM) was used as a positive control. p, phosphorylated; 0, non-phosphorylated. **c.** Immunoblot analysis of KPC cells treated with Cis (15 μM), or 5-fluorouracil (5FU, 20 μM) for 24 hours, or oxaliplatin (Oxa, 20 μM) for indicated hours. **d.** Representative TEM images showing the ultrastructure of KPC cells treated with 20 μM Cis for indicated times. The arrows point to ER. **e.** Immunoblot analysis of mitochondria-related proteins in KPC cells treated with Cis at various time points, representative of two independent biological repeats. The relative density of protein is shown below the blots. All values are presented as mean ± SEM. Statistical significance was determined using an unpaired, two-tailed Student’s t-test (**a**). **, p < 0.01; ****, p < 0.0001.

**Figure S3. IRE1α suppresses STING and its signaling in PDAC. a. Quantification of STING protein levels in wildtype and IRE1**α **KO PDAC cells. b-c.** Immunoblot analysis in HEK293T cells transfected with IRE1α and cGAS plasmids **(b)** or with IRE1α and STING plasmids **(c).** Data are representative of 2-3 independent repeats. **d.** Immunoblot analysis in HEK293T cells transfected with IRE1α and STING plasmids, followed by Cis (20μM) treatment for 24 hours. Data are representative of 3 independent repeats. **e.** Immunoblot analysis in HEK293T cells transfected with IRE1α and STING plasmids for 24 hours, followed by treatment with cycloheximide (CHX, 50 μg/mL), chloroquine (CHL, 100 μM), or MG132 (25 μM) for 6 hours. Data are representative of 2 independent repeats. The relative intensity of proteins or the phosphorylated-to -total protein ratio (p/t) is shown below the blots. All values are presented as mean ± SEM. Statistical significance was determined using an unpaired, two-tailed Student’s t-test (**a**). **, p < 0.01.

**Figure S4. IRE1α regulates STING independently of cGAS or XBP1s. a.** Immunoblot analysis following immunoprecipitation of exogenous cGAS-FLAG from HEK293T cells transfected with cGAS and IRE1α plasmids. IP, immunoprecipitation. Data are representative of 2 independent repeats. **b.** Immunoblot analysis of STING, IRE1α, QRICH1 and EIF2α following immunoprecipitation of exogenous STING-FLAG from HEK293T cells transfected with STING and IRE1α plasmids**.** IgG, immunoglobulin G. * indicates the target band. Data are representative of two independent repeats. **c.** q-PCR analysis of indicated genes in IRE1α^-/-^ and XBP1s-overexpressing (OE) IRE1α^-/-^ KPC cells treated with vehicle or 20 μM Cis for 24 hours (n = 9, combined from three independent repeats. **d.** Immunoblot analysis of *WT* and *Hrd1^-/-^* KPC cells treated with vehicle control or 20μΜ Cis, followed by CHX for indicated durations. Representative images from two independent biological repeats are shown. All values are presented as mean ± SEM. Statistical significance was determined using an unpaired, two-tailed Student’s t-test (**c**). **, p < 0.01; ****, p < 0.0001; n.s., not significant.

**Figure S5. Ribosomal sequencing of vehicle or Cis treated KPC cells. a.** Quality of Ribosomal Sequencing Data: Proportion of in-frame reads (aligned within the standard codon reading frame) among total reads, with +1/-1 Frame indicating a one-nucleotide shift. **b.** This bar chart shows the number of reads mapped to three regions of the transcript: 5' UTR, 3' UTR, and CDS. **c.** Length Distribution of Ribosome Footprints (RFPs): Histogram showing the length distribution of RFPs. When RFPs are 29-30 nt in length, the ribosomal cleavage is more efficient. **d-e.** Read Distributions Near Start and Stop Codons: Aggregation plots show the distributions of ribosome footprint reads around the start (d) and stop (e) codons. Normal ribosomal translation produces a peak every three positions, reflecting the triplet codon periodicity.

**Figure S6. Cisplatin treatment suppresses protein translation.**

**a-b.** Immunoblot analysis of KPC cells (**a**) and Panc02 cells (**b**) treated with Cis ± puromycin for the indication durations. Representative images from two independent biological repeats are shown.

**Figure S7. ER stress inducer sensitize cancer cells to chemotherapy.** **a-b.** q-PCR (**a**) and Immunoblot (**b**) analysis of KPC cells treated with 20 μM Cis, Tunicamycin (TM, 5μg/ml), or their combination for 24 hours (n = 6 for **a**, combined from two independent repeats). **c.** Schematic representation of the experimental cancer model in which wildtype Panc02 cells-transplanted immunodeficient Nude mice received intraperitoneal (i.p.) injections of Chemo drugs (Cis, 3 mg kg^−1^, 5FU, 25 mg kg^−1^, Irinotecan, 25 mg kg^−1^), either alone or combined with peritumoral injections of TM (0.3 mg kg^−^1) with or without H151 (5 mg kg^−1^). Representative images of pancreatic tumors and quantitation of tumor weights at the end of experiment (n = 5, 4, 4, 6 mice per group, left to right, Data are representative of two biological repeats). All values are presented as mean ± SEM (a, c). Statistical significance was determined using an unpaired, two-tailed Student’s t-test. *, p < 0.05; **, p < 0.01; ***, p < 0.001; ****, p < 0.0001.
