# Supplementary figures and images for: IRE1α translational suppression potentiates STING-dependent chemoresistance in pancreatic cancer
Source: Cell Death Dis. 2025 Oct 6;16(1):680. doi: 10.1038/s41419-025-07999-x (PMC12501023; doi:10.1038/s41419-025-07999-x)

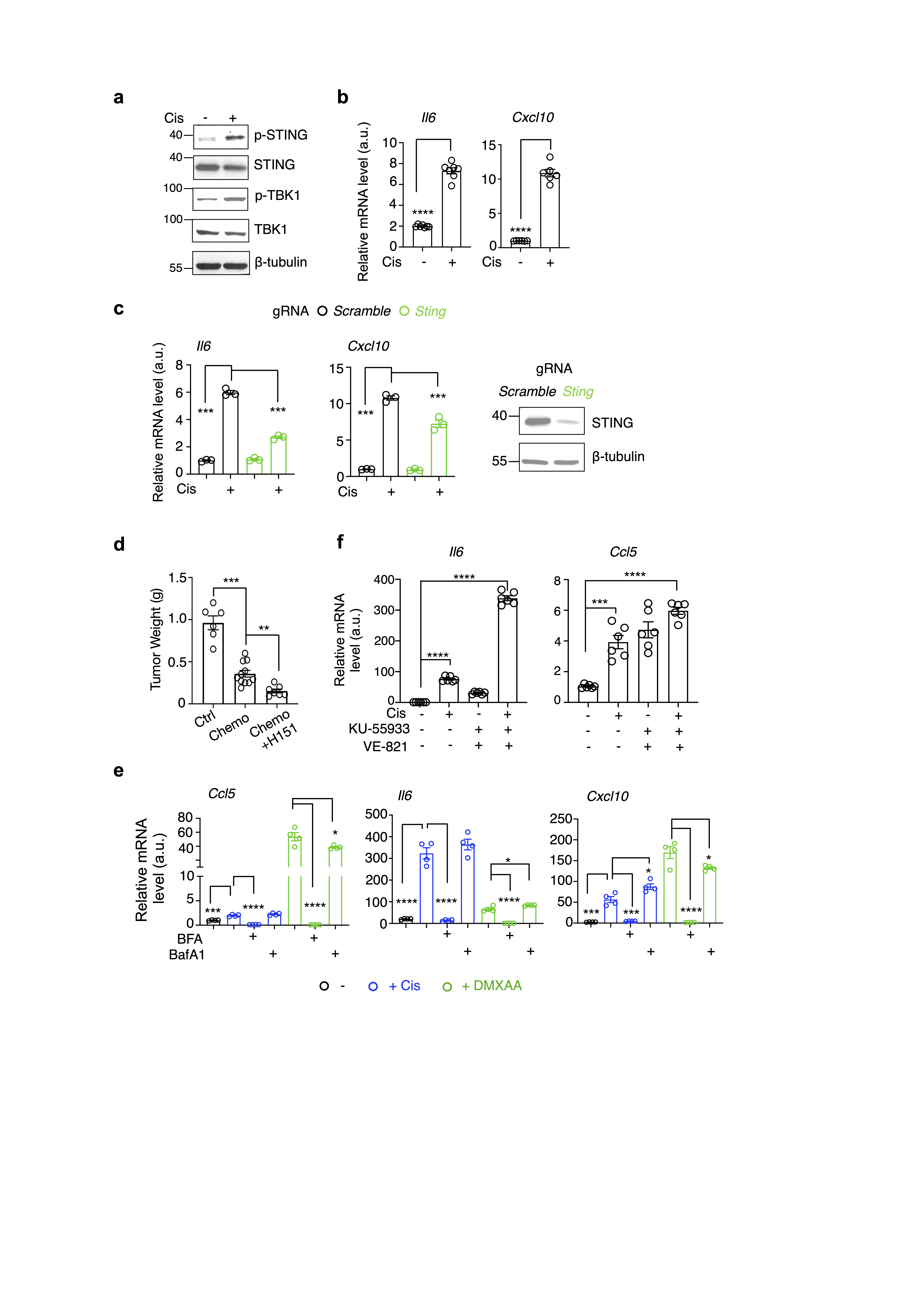

Supplement: Supplementary file 2 — Figure S1 [file 41419_2025_7999_MOESM2_ESM.jpg]

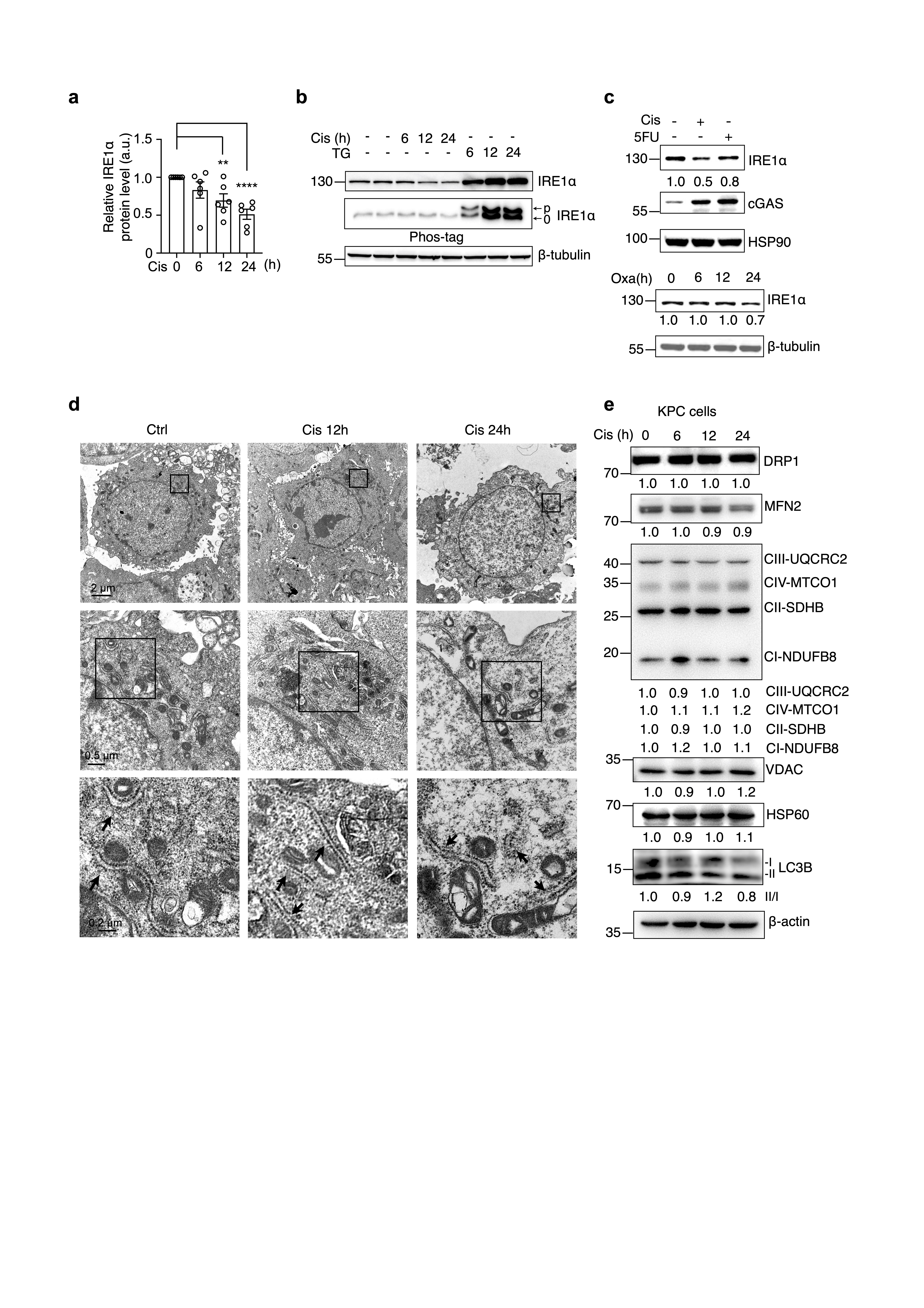

Supplement: Supplementary file 3 — Figure S2 [file 41419_2025_7999_MOESM3_ESM.jpg]

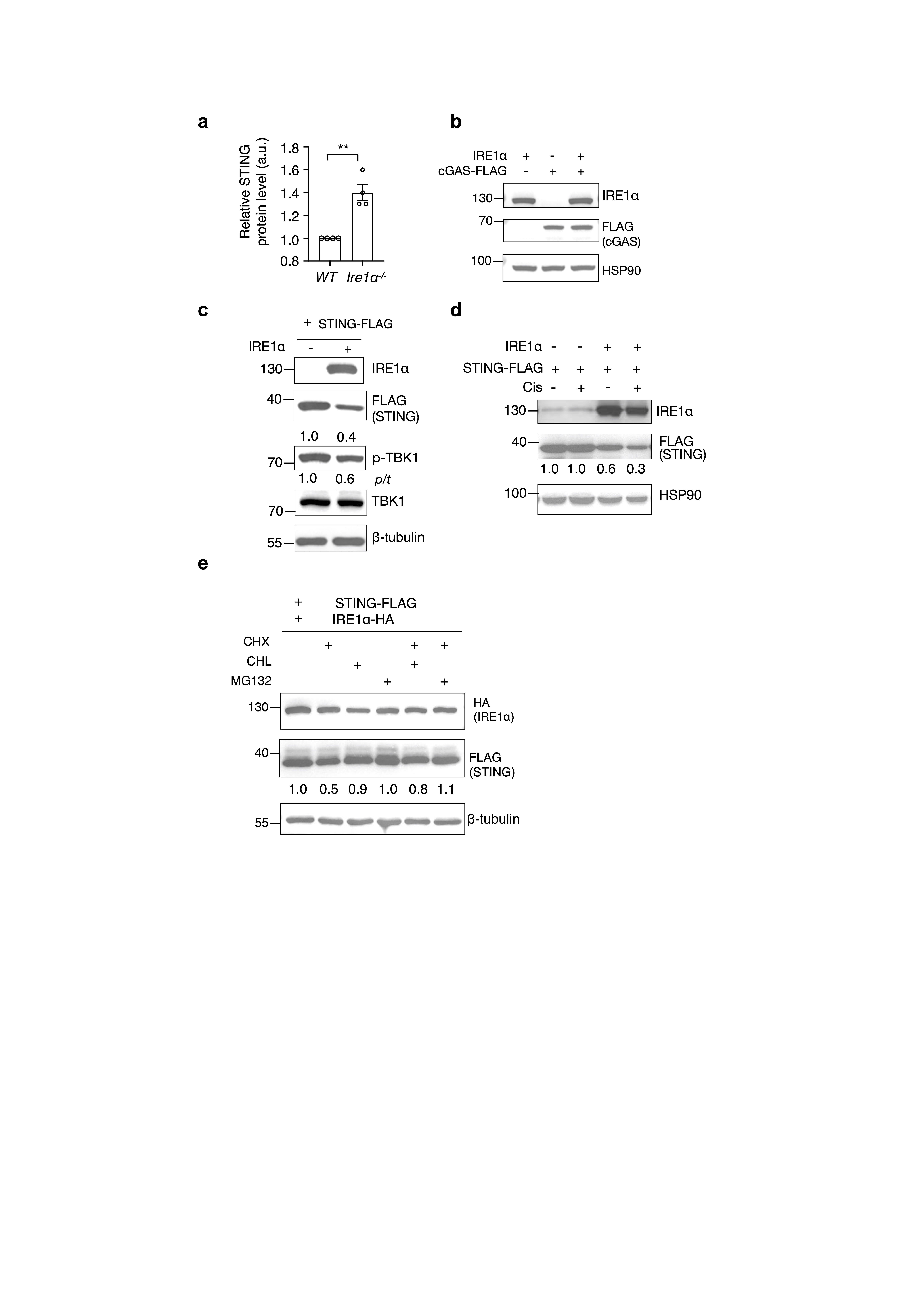

Supplement: Supplementary file 4 — Figure S3 [file 41419_2025_7999_MOESM4_ESM.jpg]

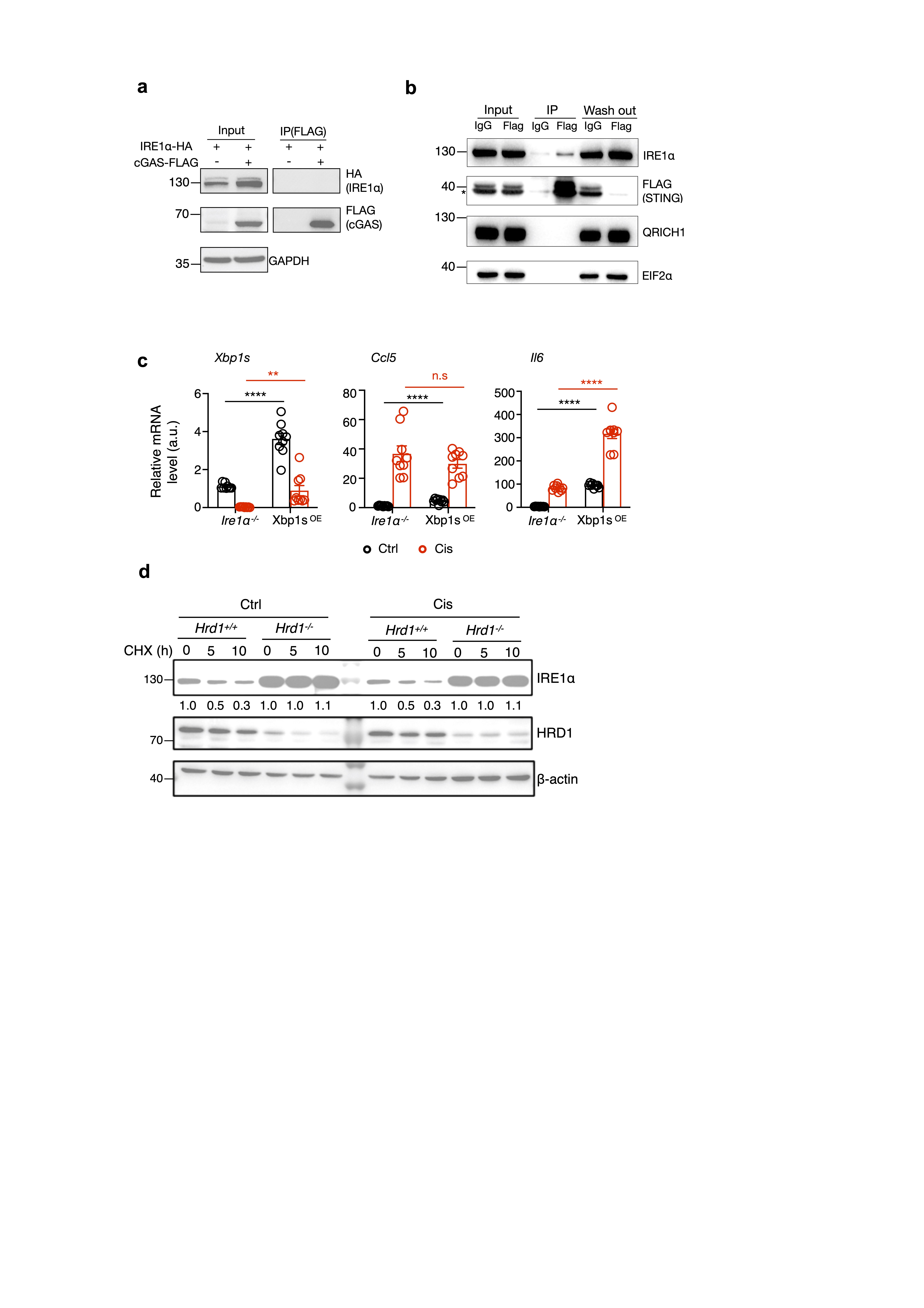

Supplement: Supplementary file 5 — Figure S4 [file 41419_2025_7999_MOESM5_ESM.jpg]

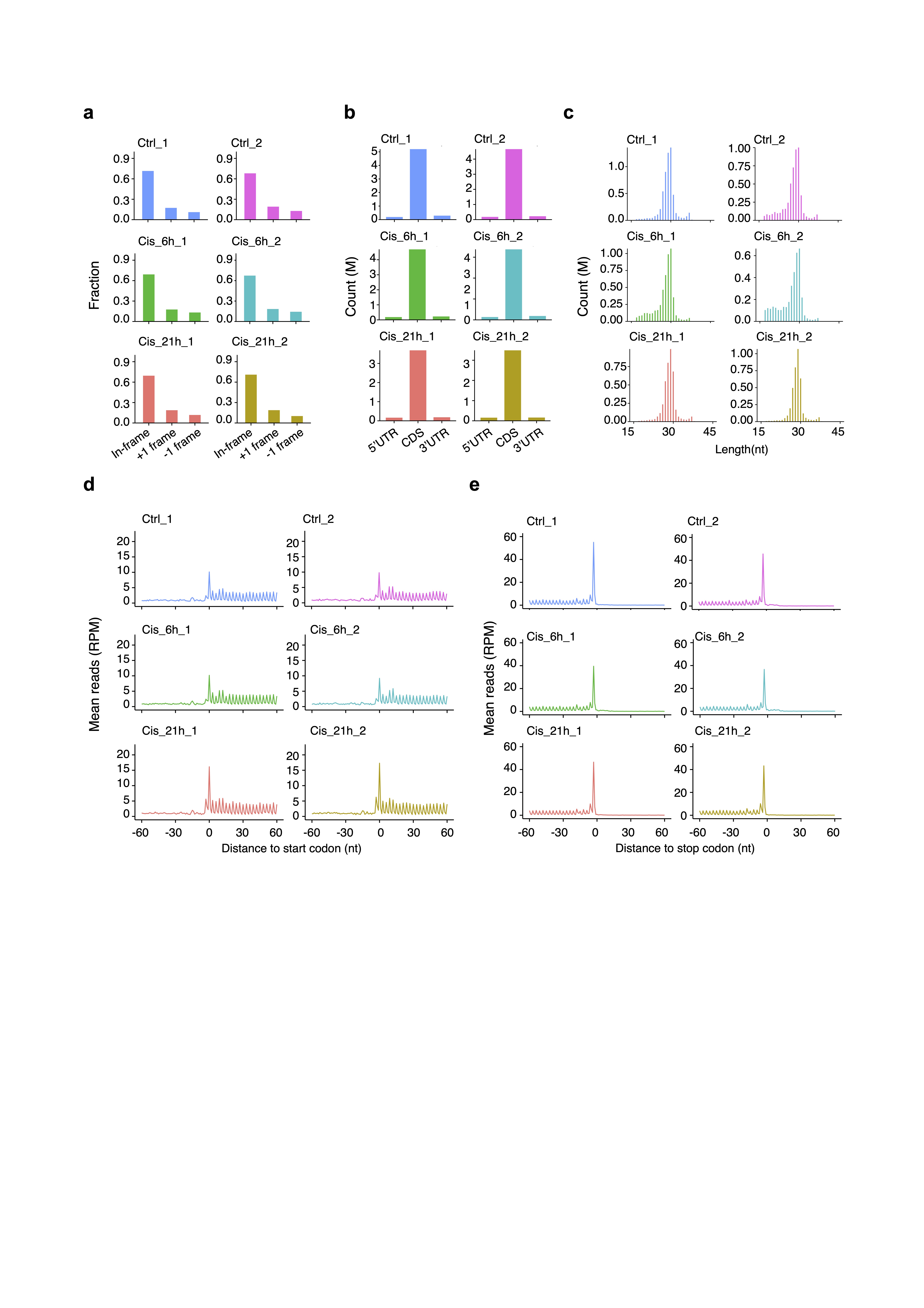

Supplement: Supplementary file 6 — Figure S5 [file 41419_2025_7999_MOESM6_ESM.jpg]

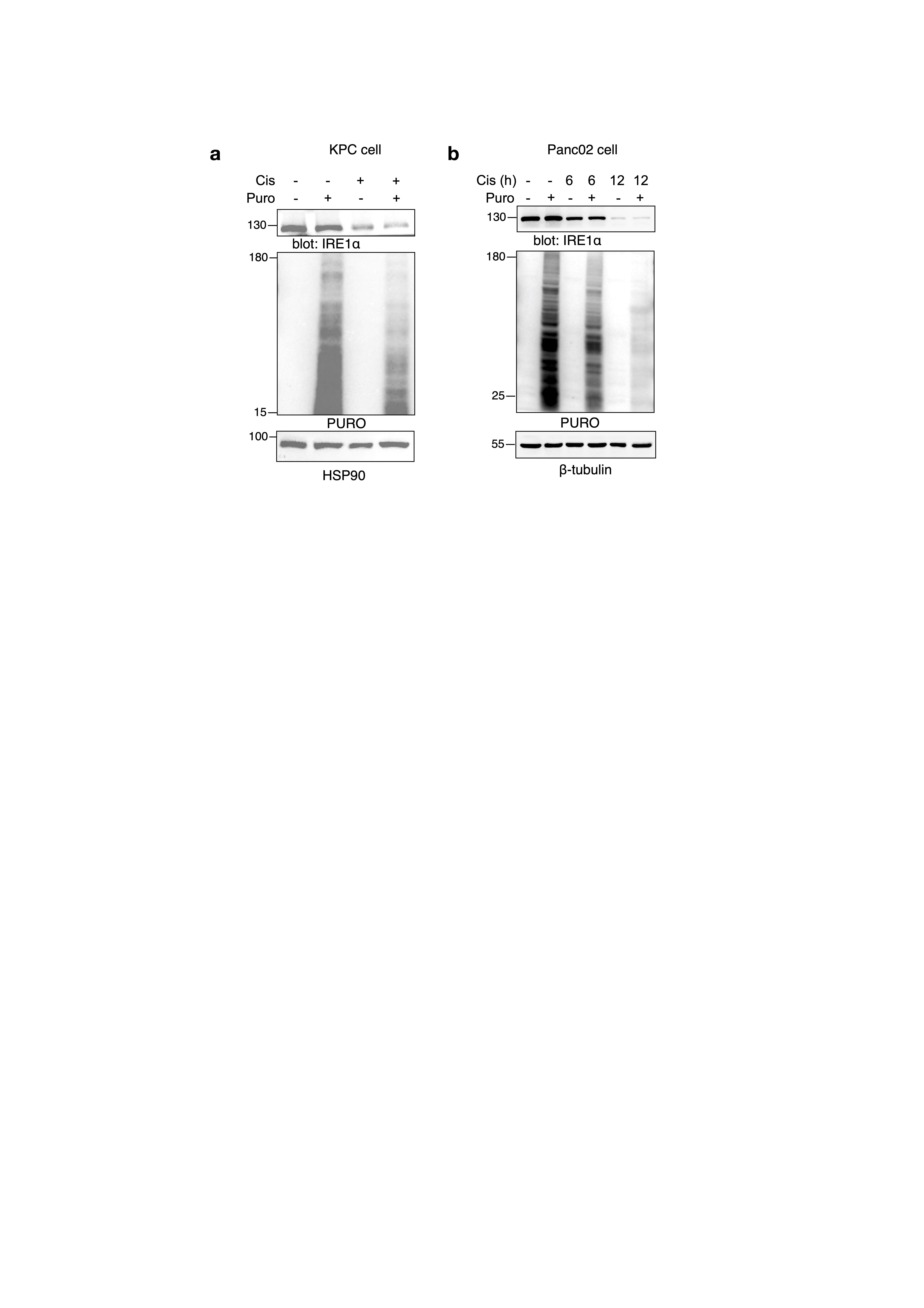

Supplement: Supplementary file 7 — Figure S6 [file 41419_2025_7999_MOESM7_ESM.jpg]

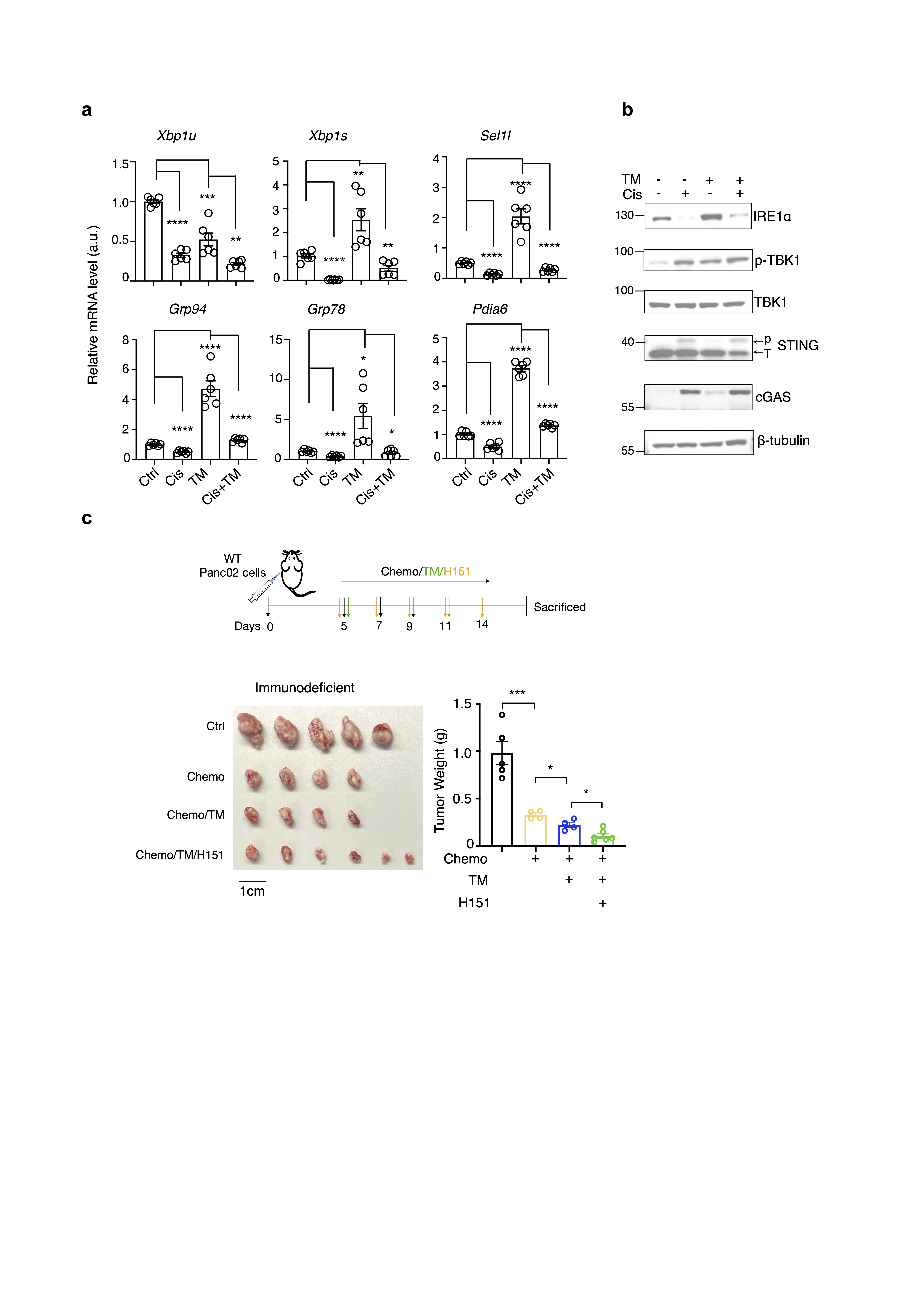

Supplement: Supplementary file 8 — Figure S7 [file 41419_2025_7999_MOESM8_ESM.jpg]
