# Supplementary material for: IRE1α translational suppression potentiates STING-dependent chemoresistance in pancreatic cancer
Source: Cell Death Dis. 2025 Oct 6;16(1):680. doi: 10.1038/s41419-025-07999-x (PMC12501023; doi:10.1038/s41419-025-07999-x)

Figure 1e

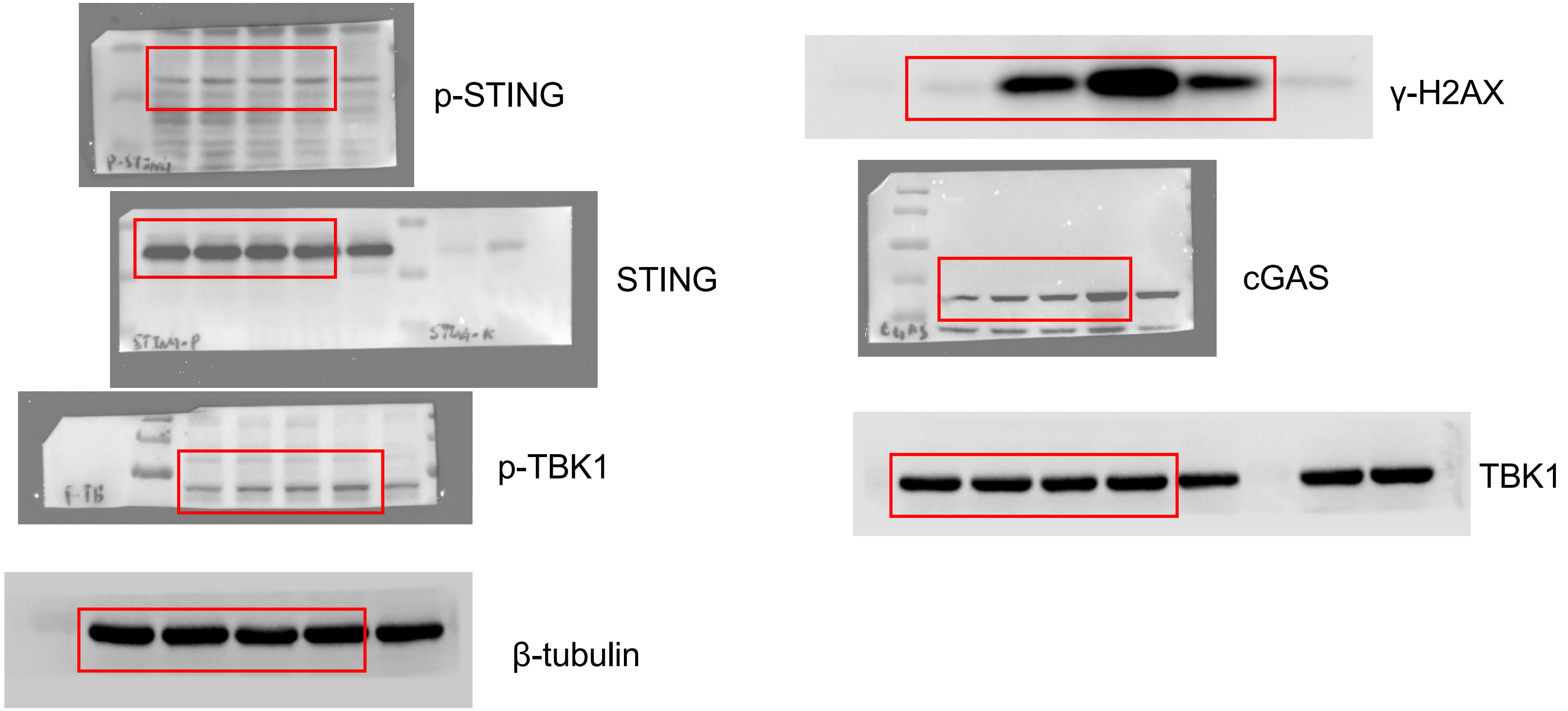

Figure 1f

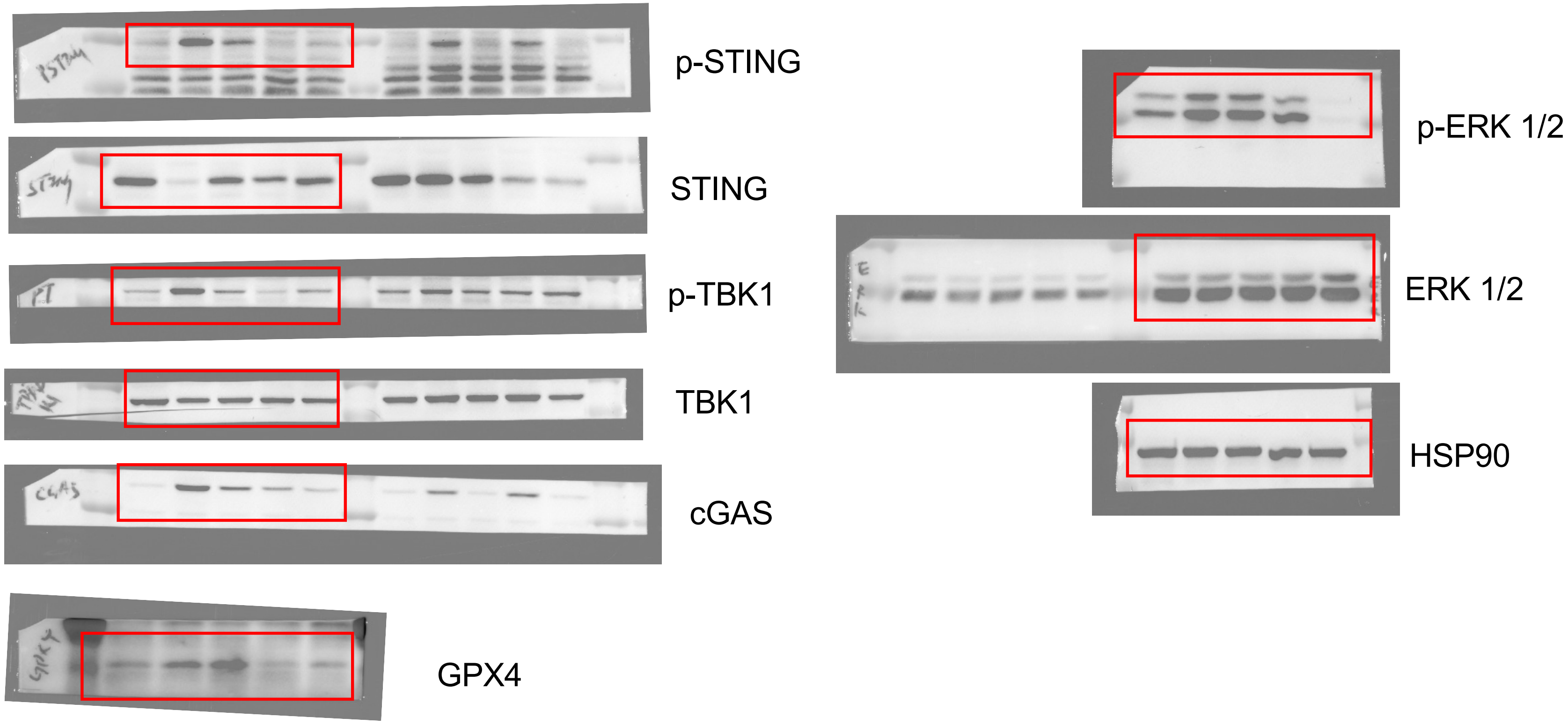

Figure 1h

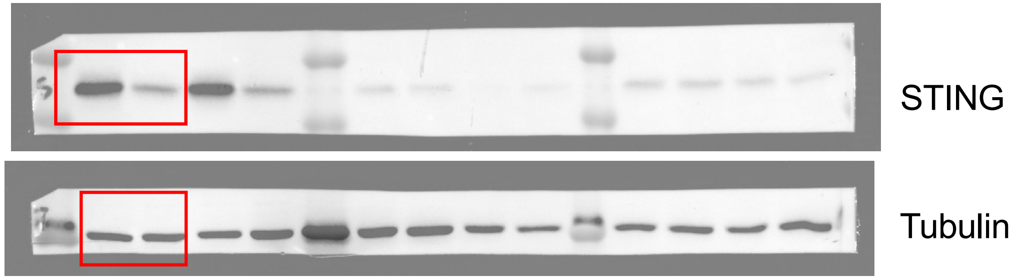

Figure 2e

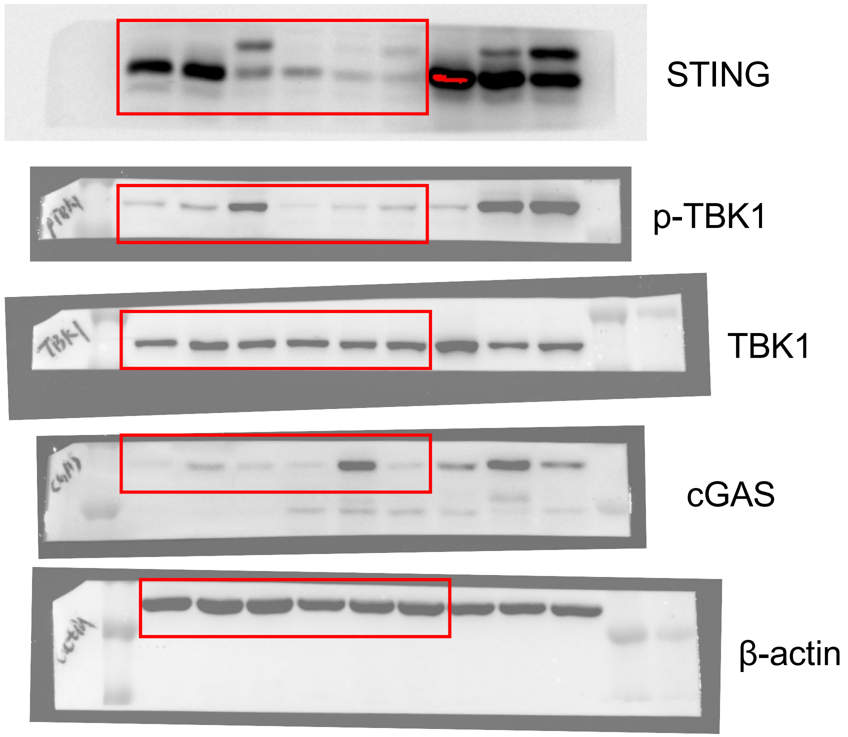

Figure 2f

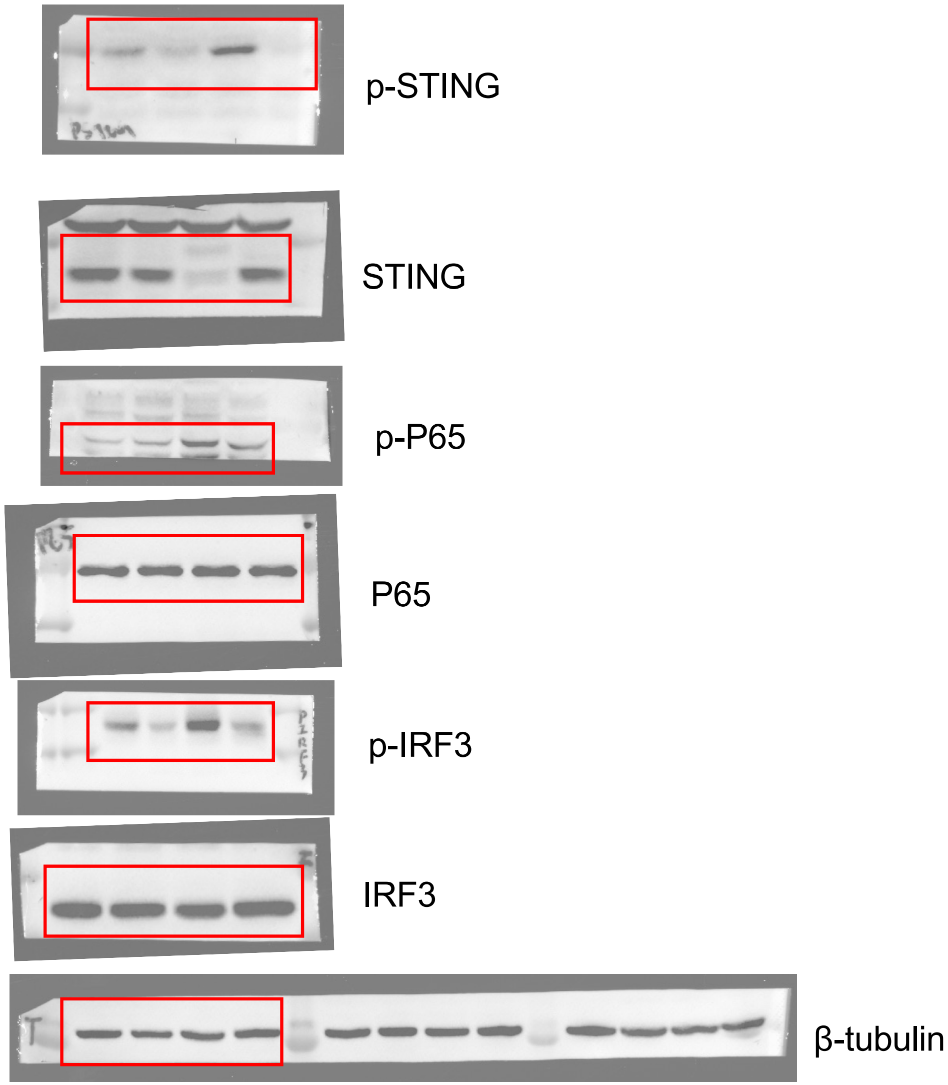

Figure 2h

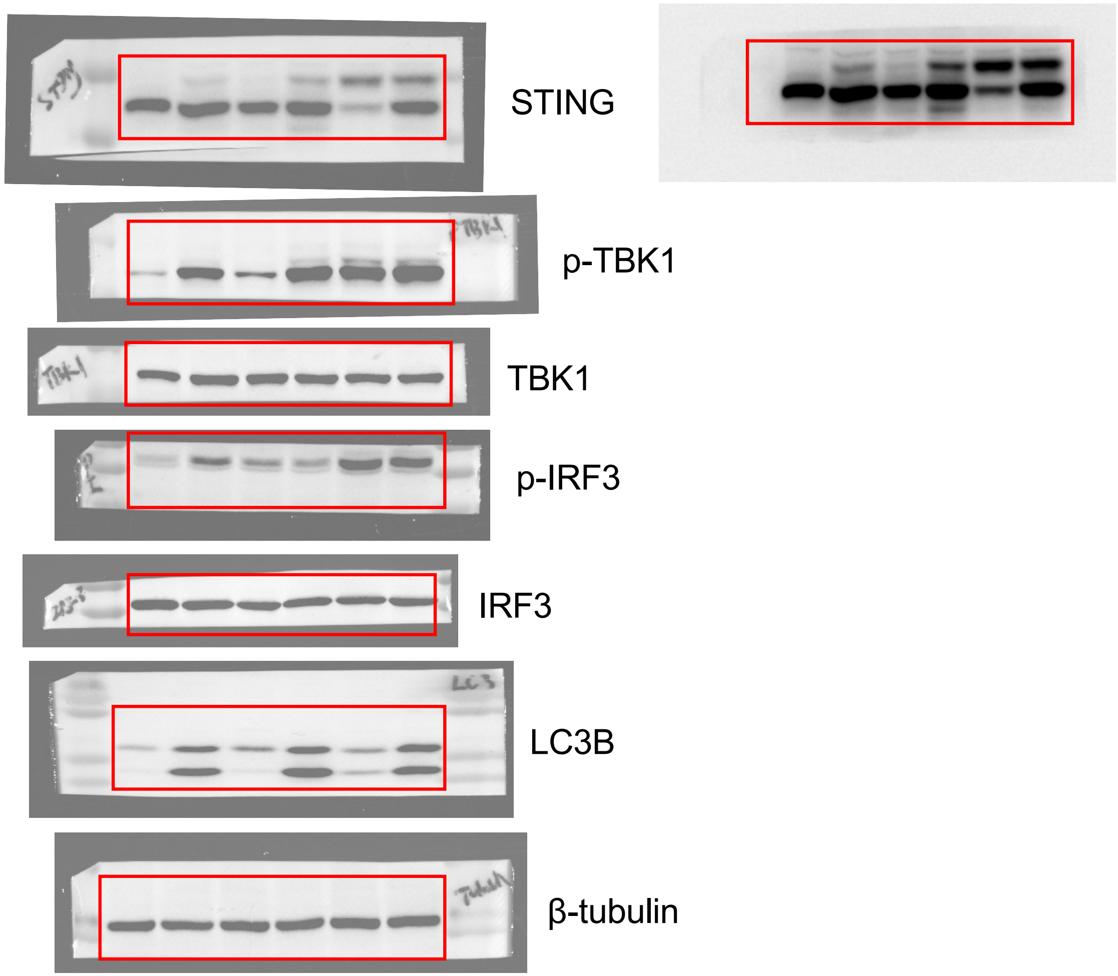

Figure 3c

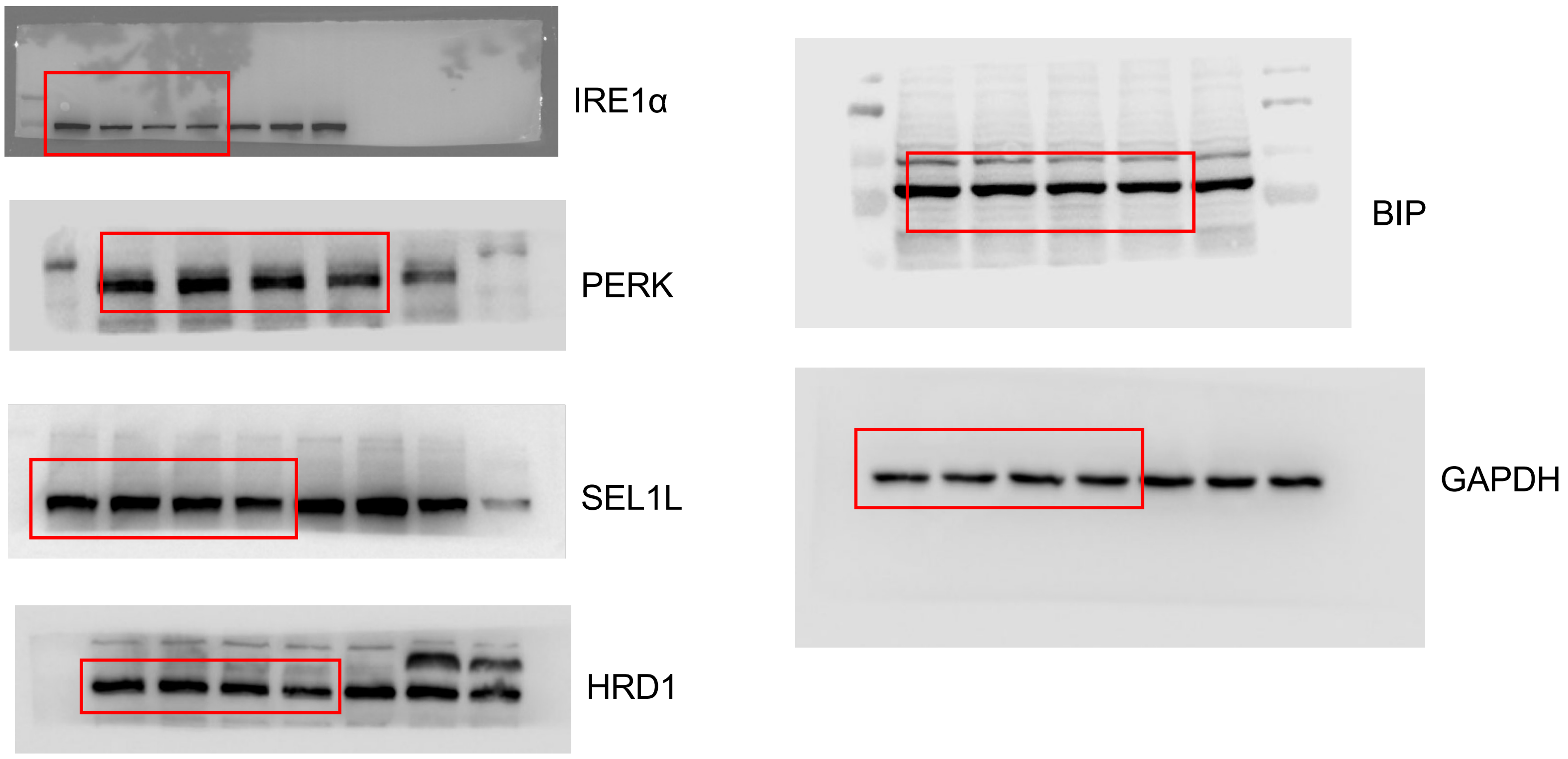

Figure 3d

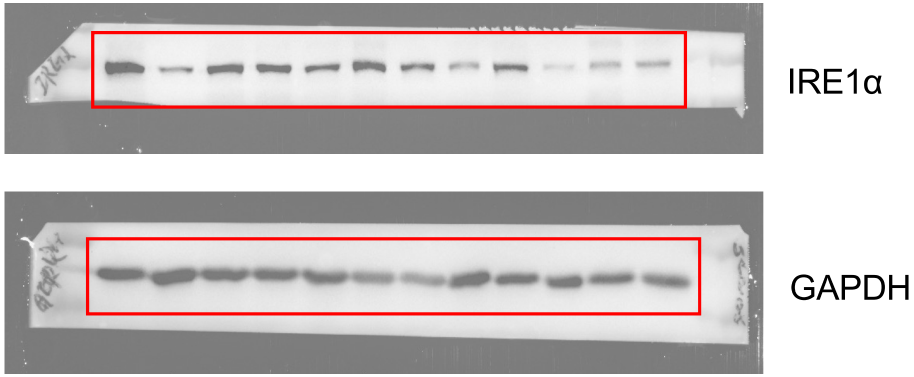

Figure 3e

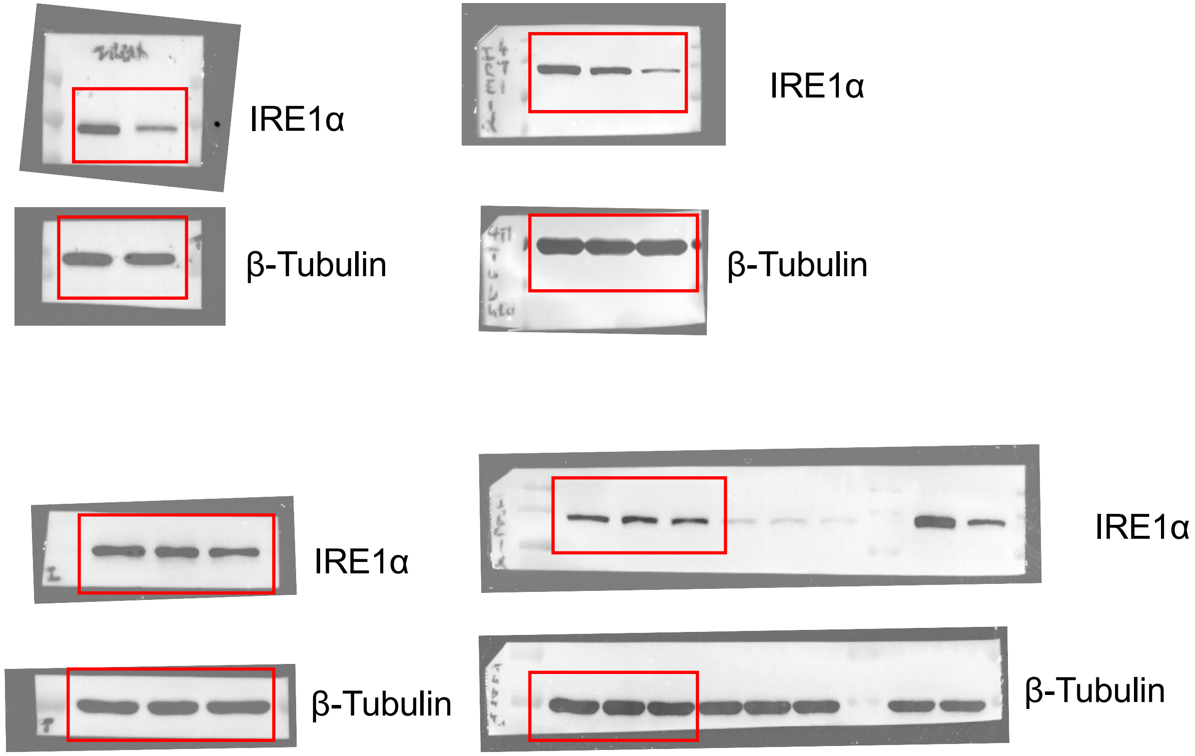
IRE1α
β-Tubulin
IRE1α
β-Tubulin
IRE1α
β-Tubulin
IRE1α
β-Tubulin

Figure 4b

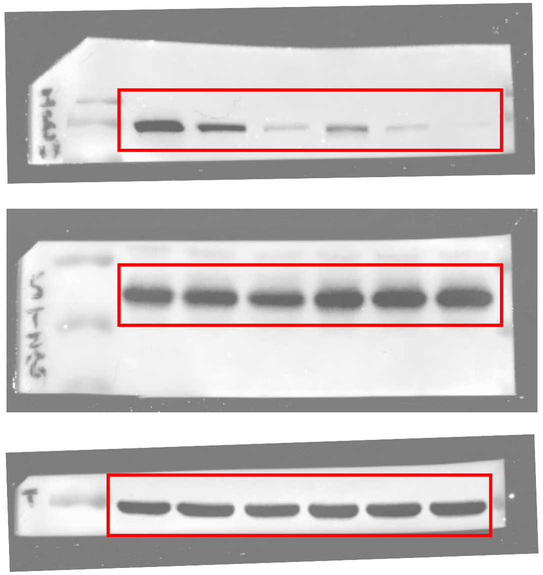

Figure 4d

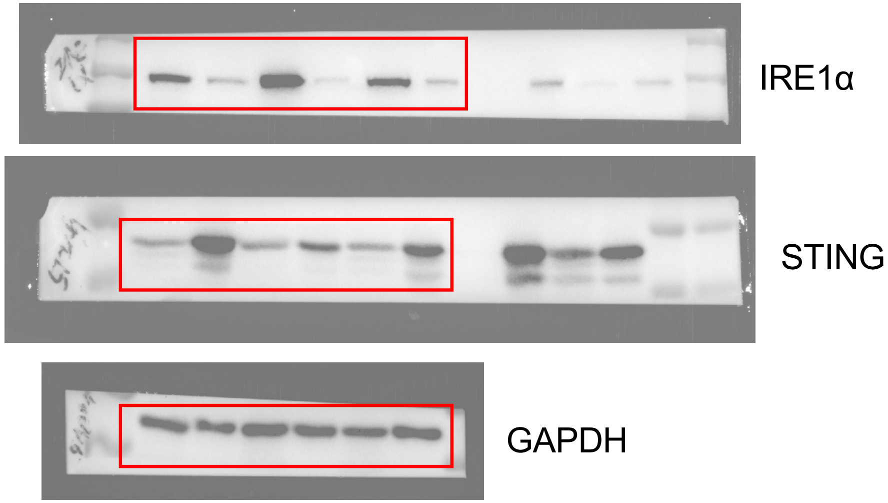

Figure 4f

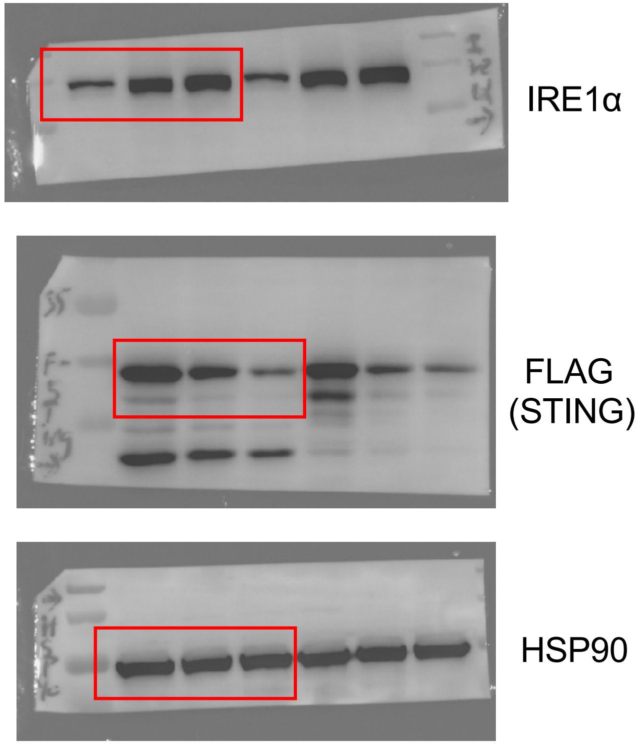

Figure 4h

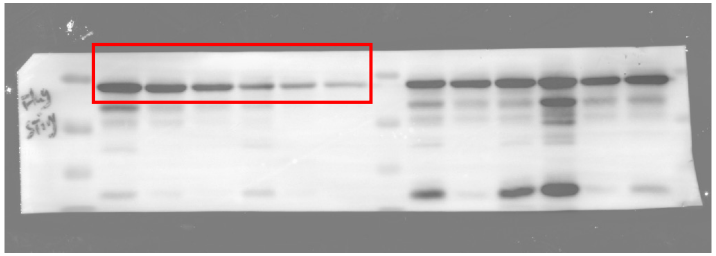

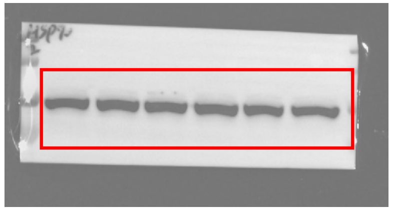

Figure 4i

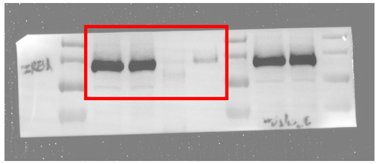

IRE1α

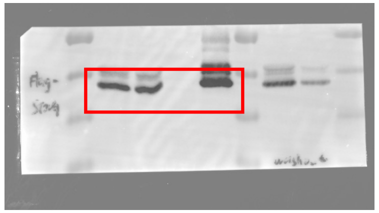

FLAG
(STING)

Figure 4k

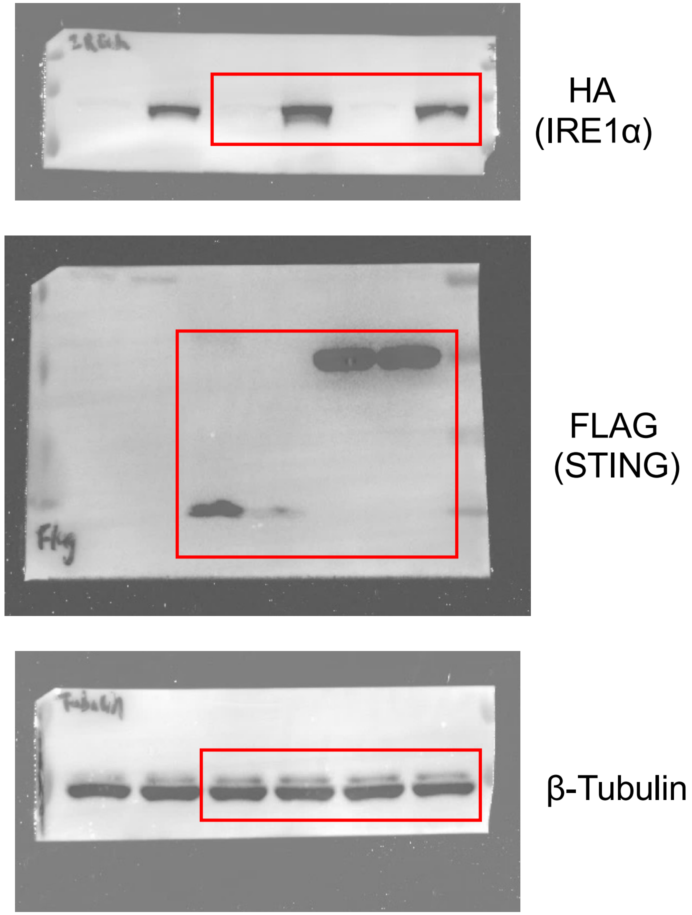

Figure 4I

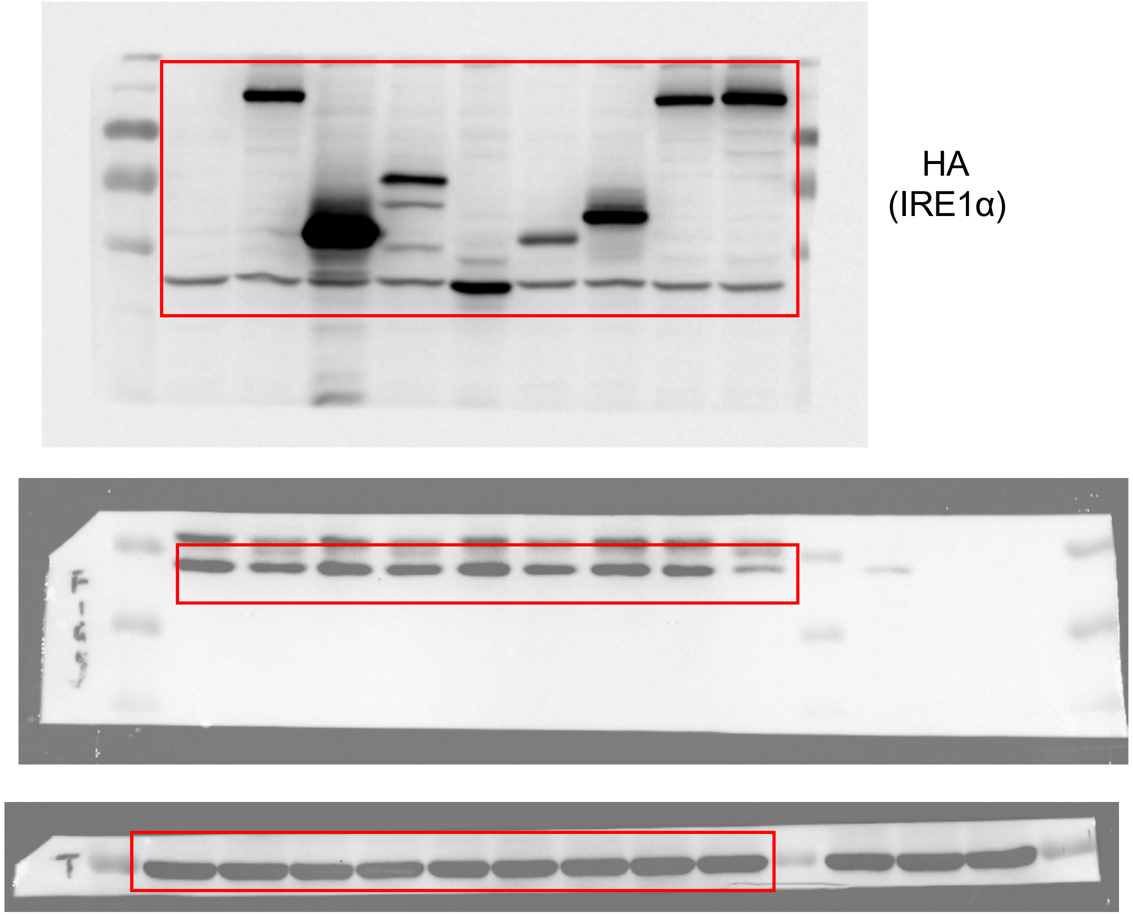

HA (IRE1α)

FLAG (STING)

β-Tubulin

Figure 5b

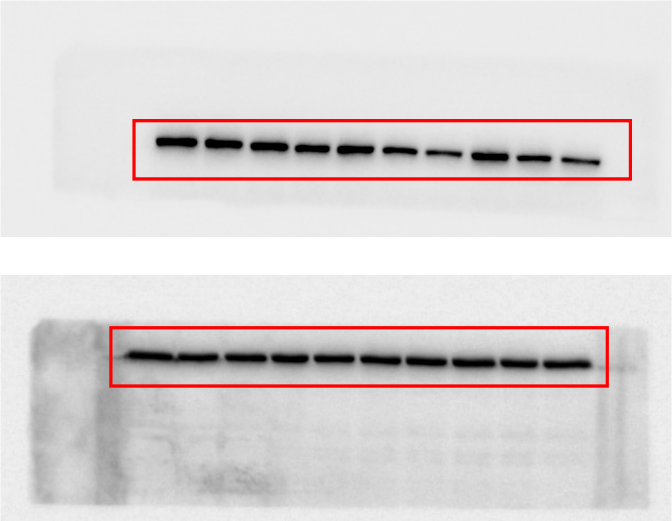

IRE1α

β-tubulin

Figure 5d

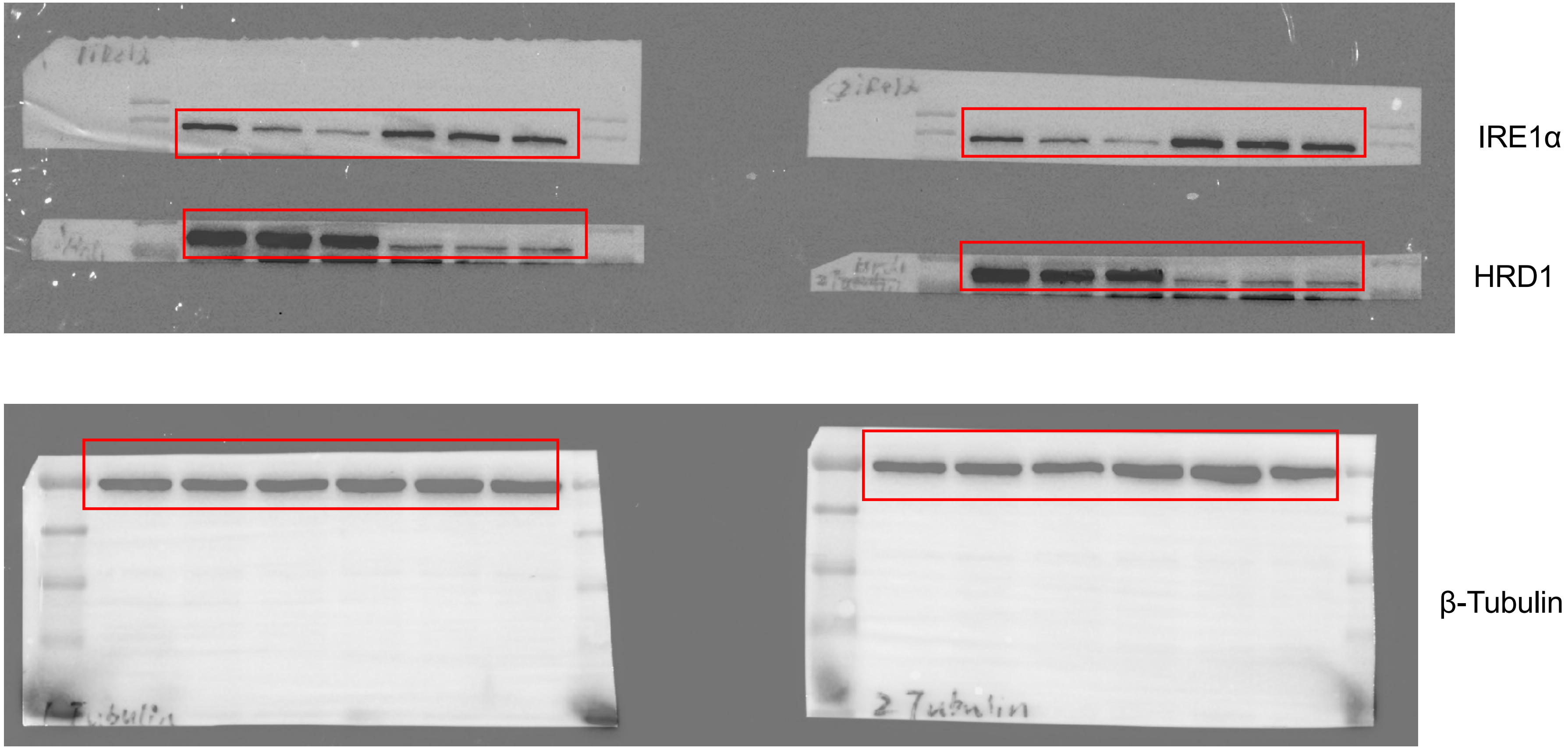

Figure 6b

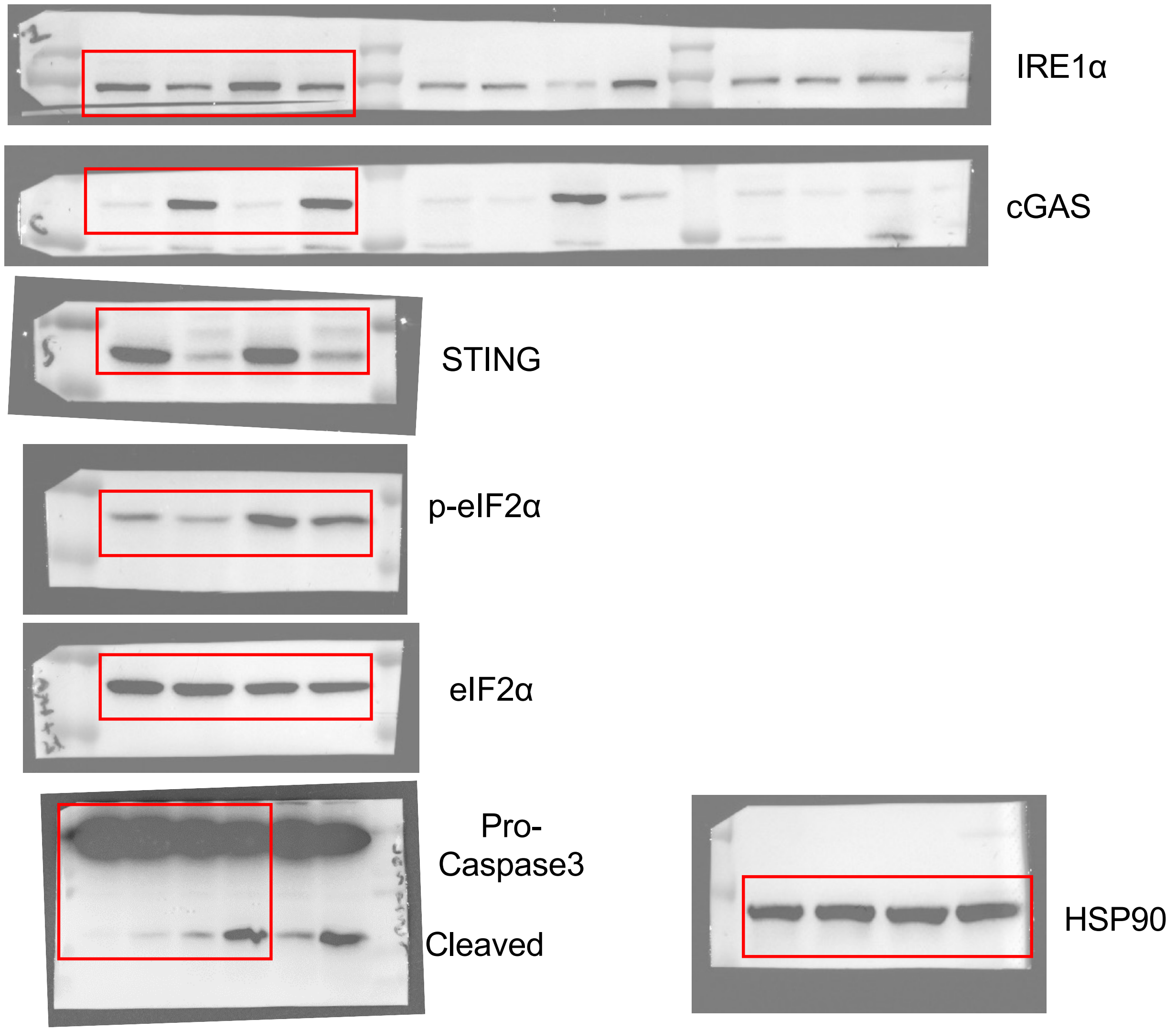

Figure S1a

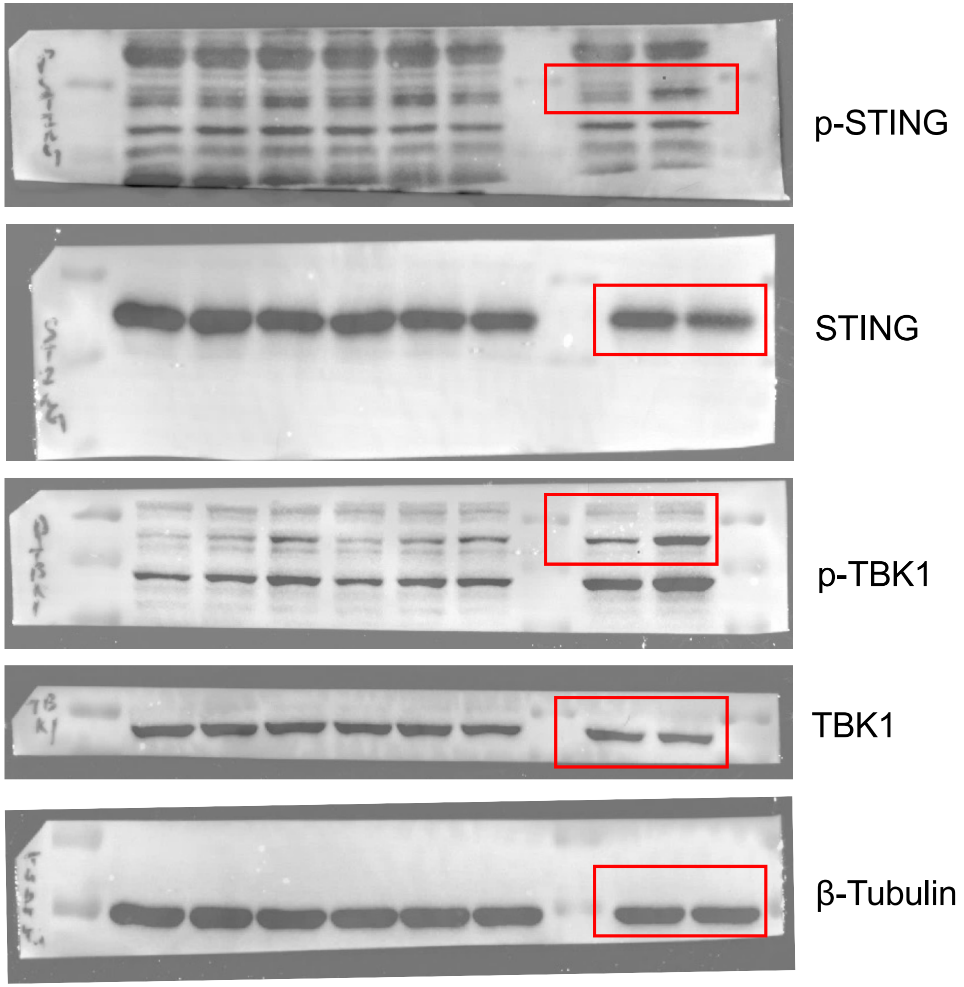

Figure S2b

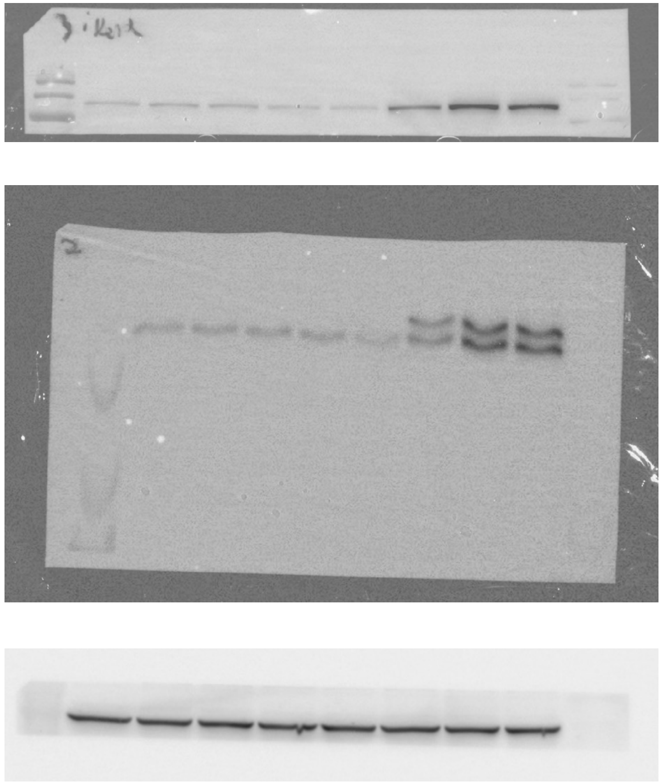

Figure S2c

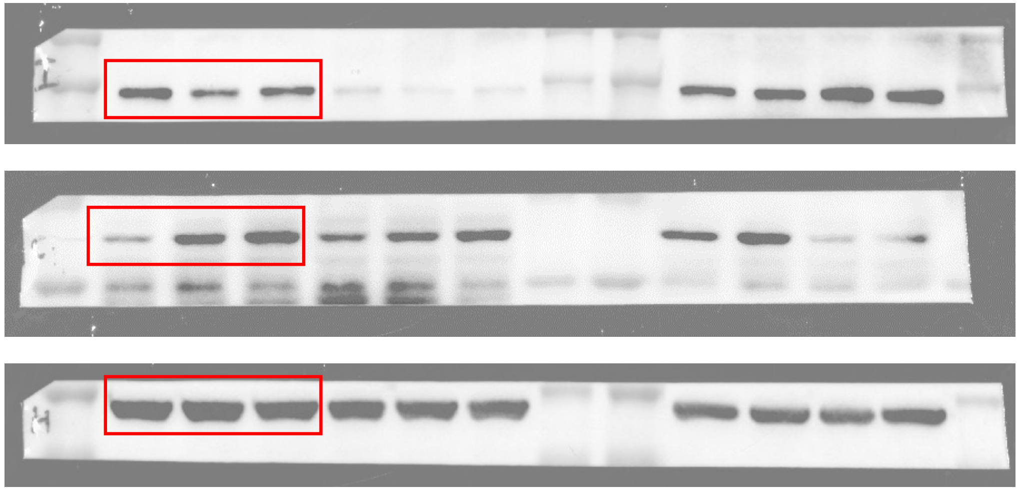

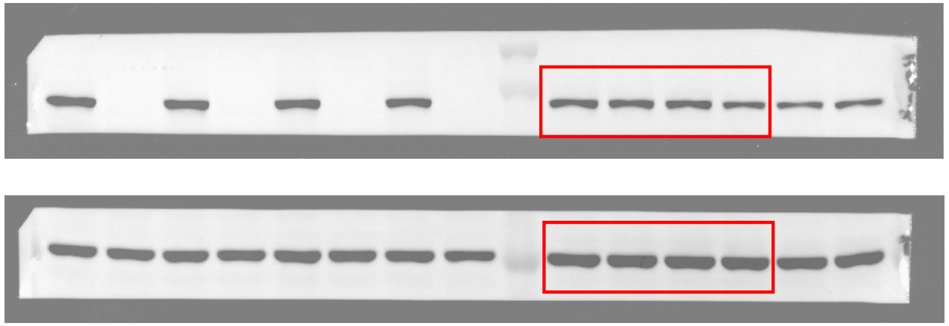

Figure S2e

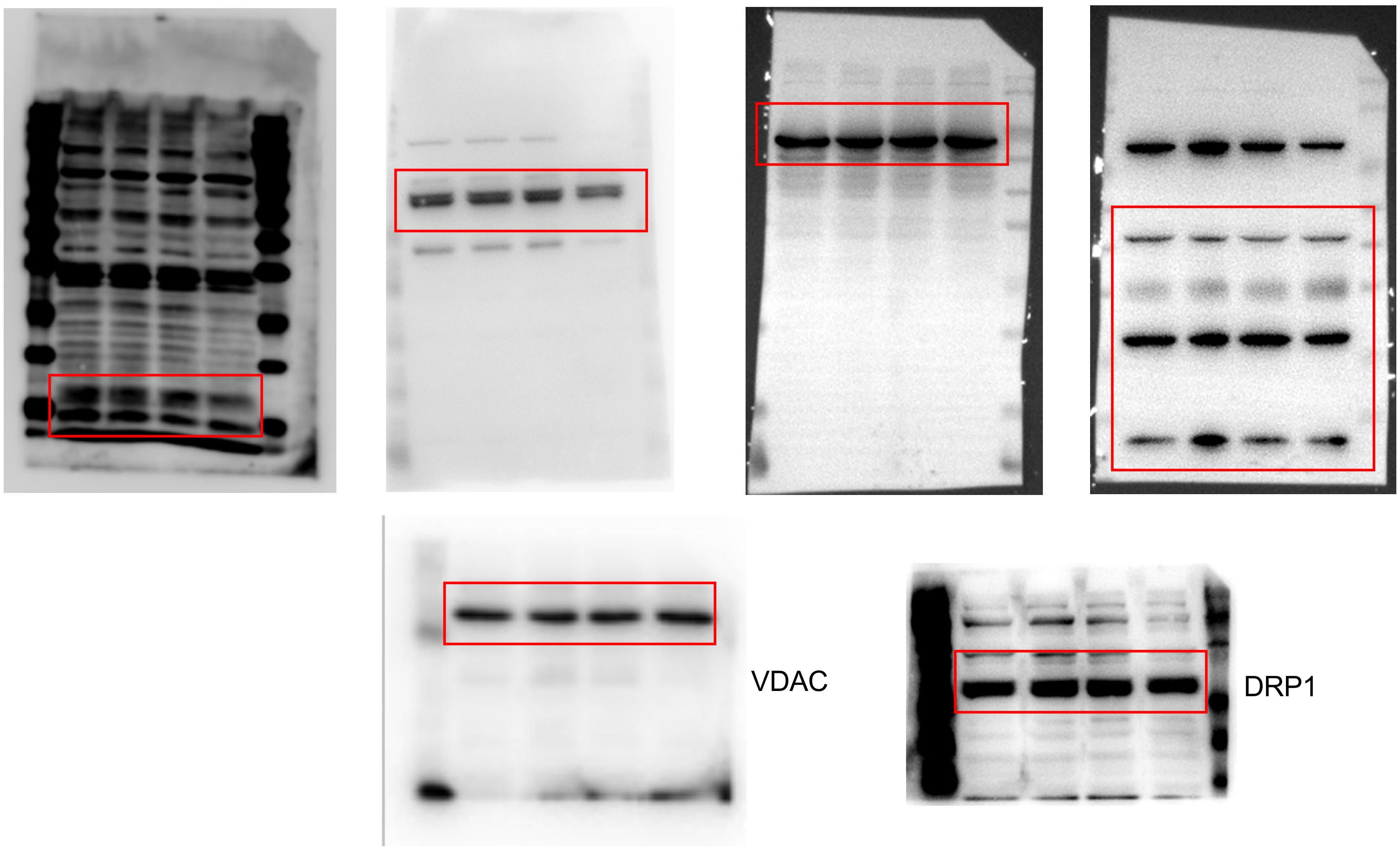
LC3B
MFN2
HSP60
CIII-UQCRC2
CIV-MTCO1
CII-SDHB
CI-NDUFB8
VDAC
DRP1

Figure S3b

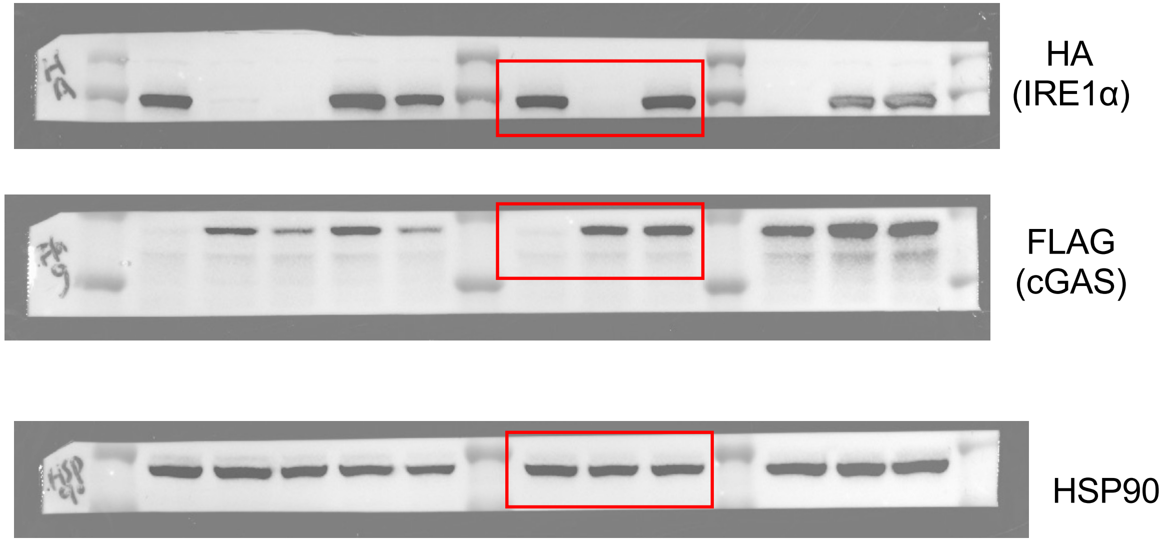

Figure S3c

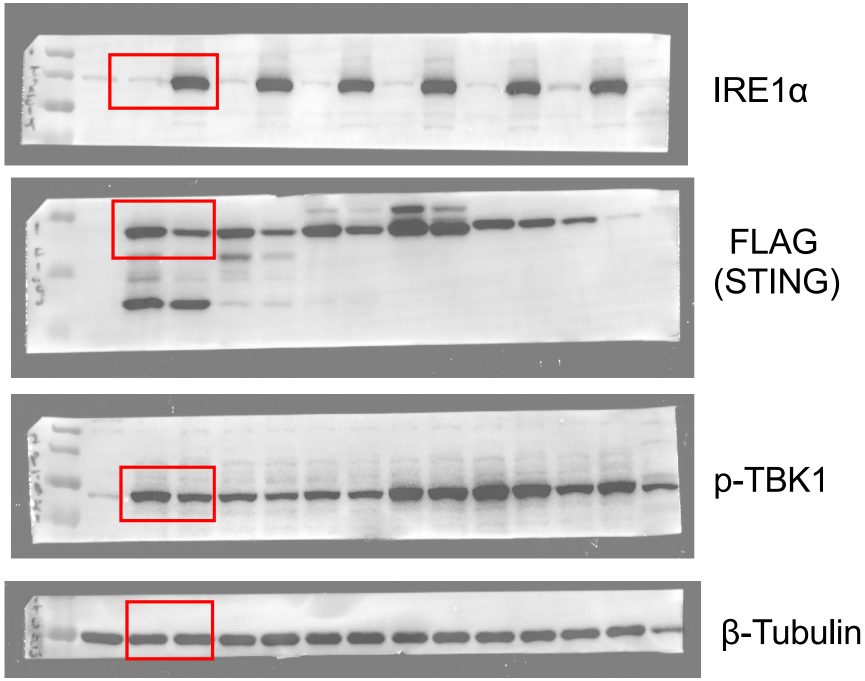

Figure S3d

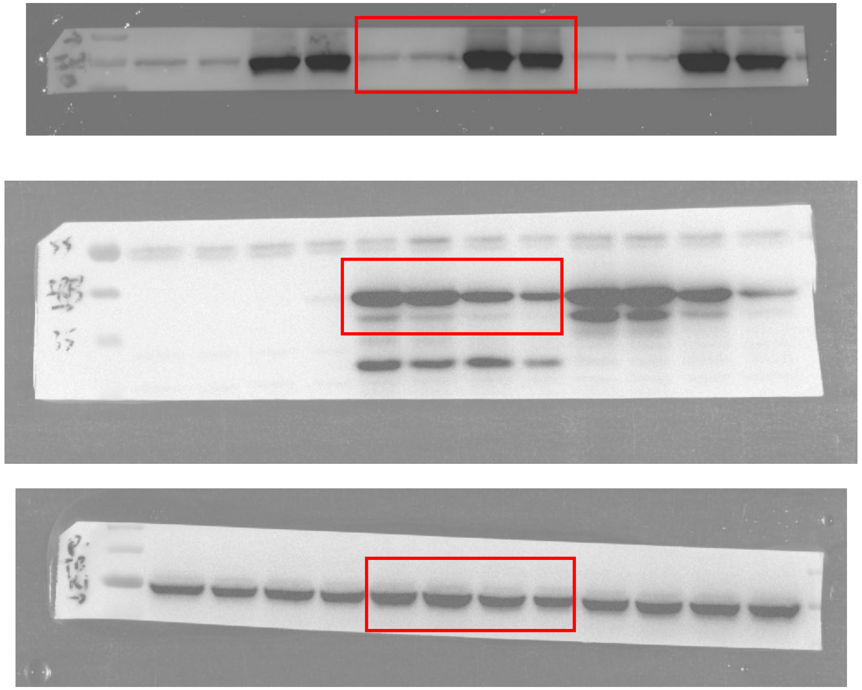

Figure S3e

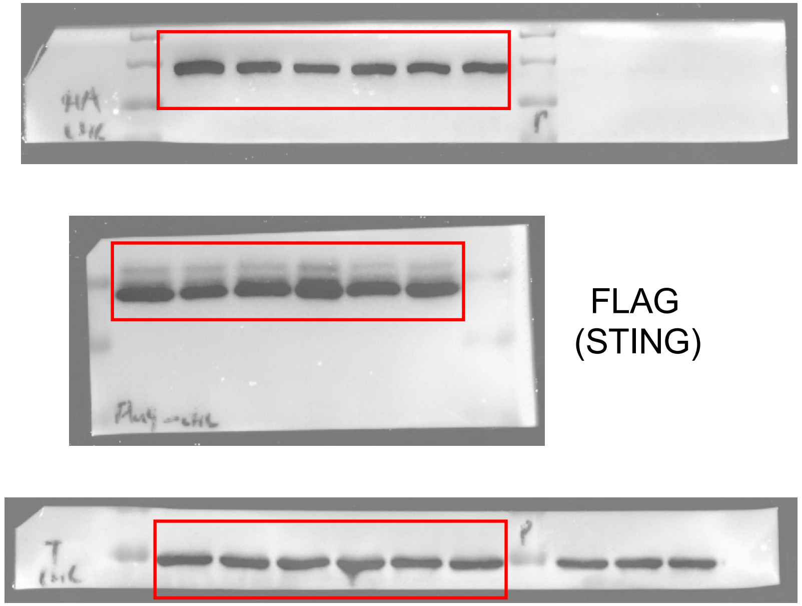

Figure S4a

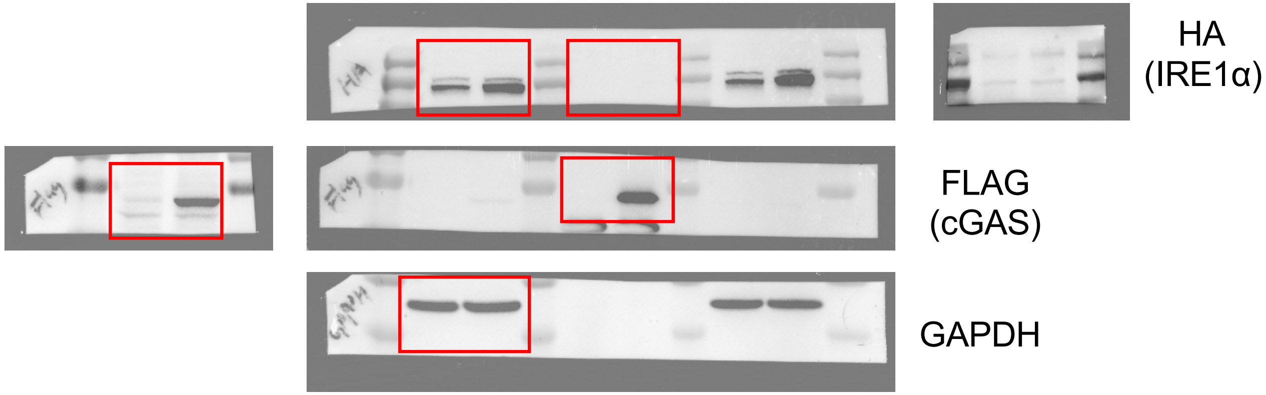

Figure S4b

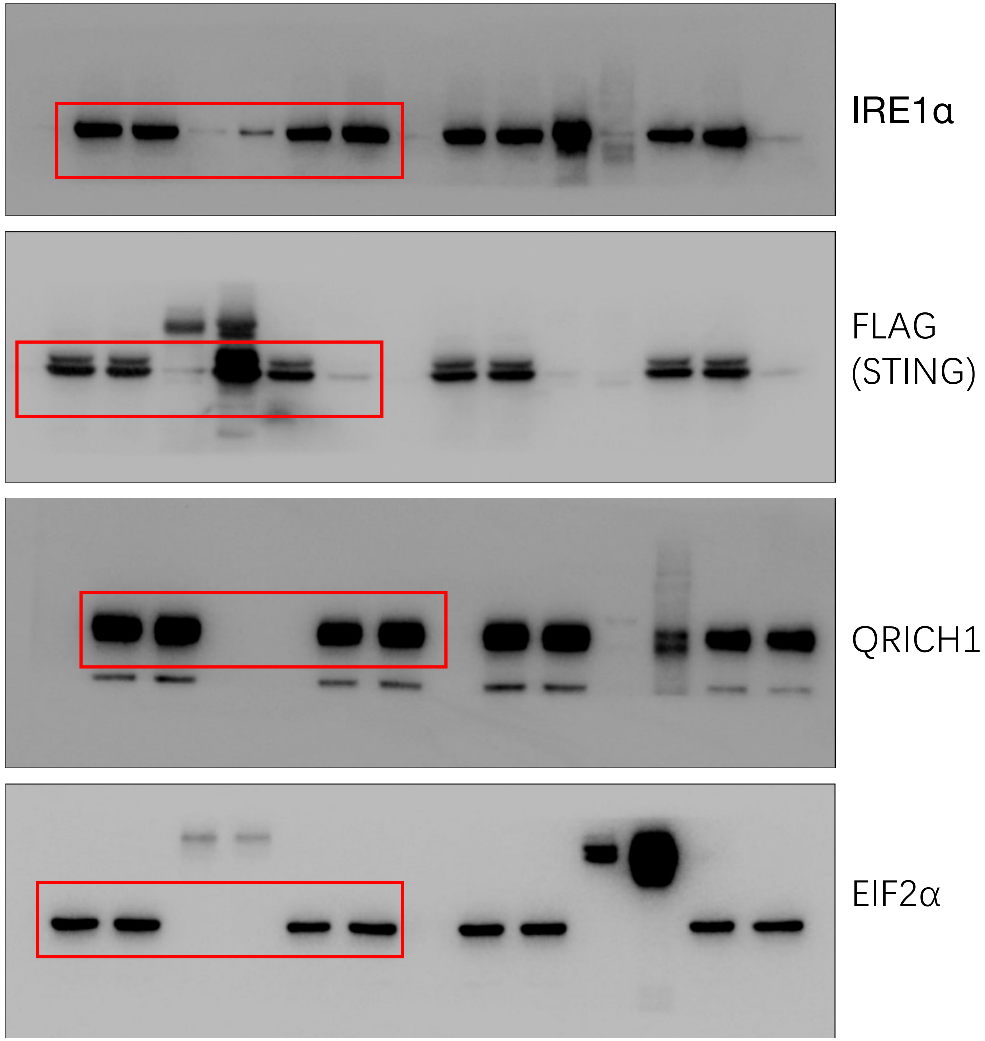

Figure S4d

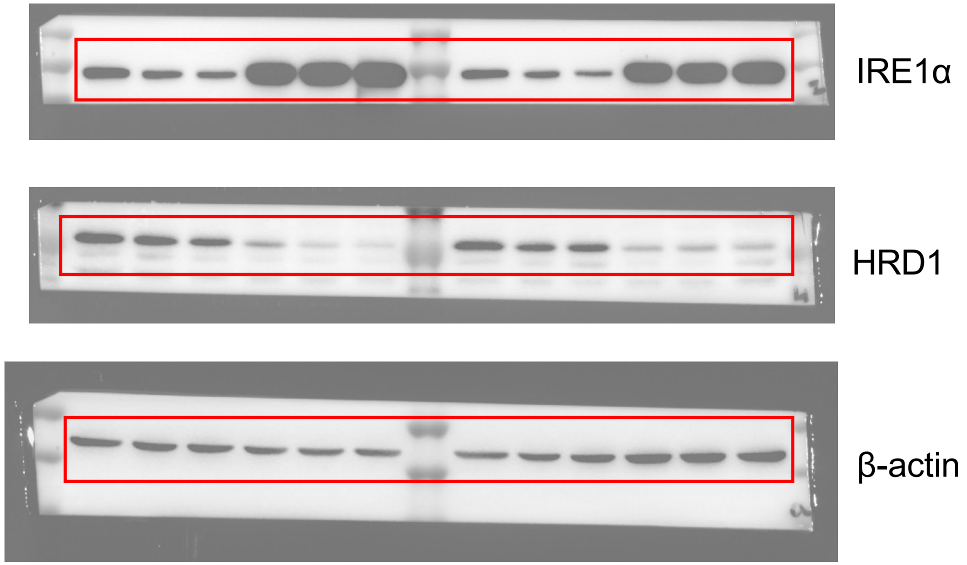

Figure S6a

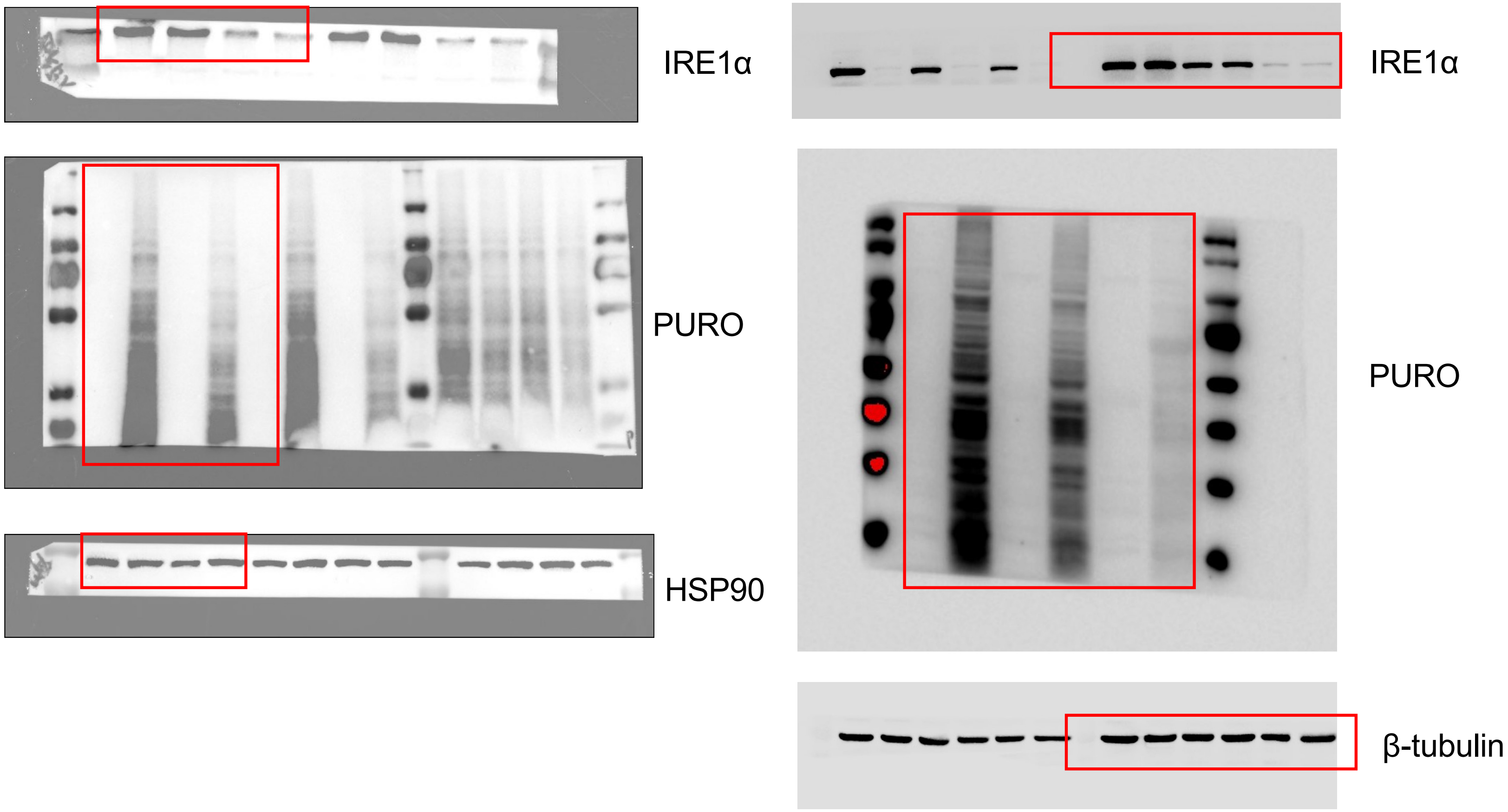

Figure S7b

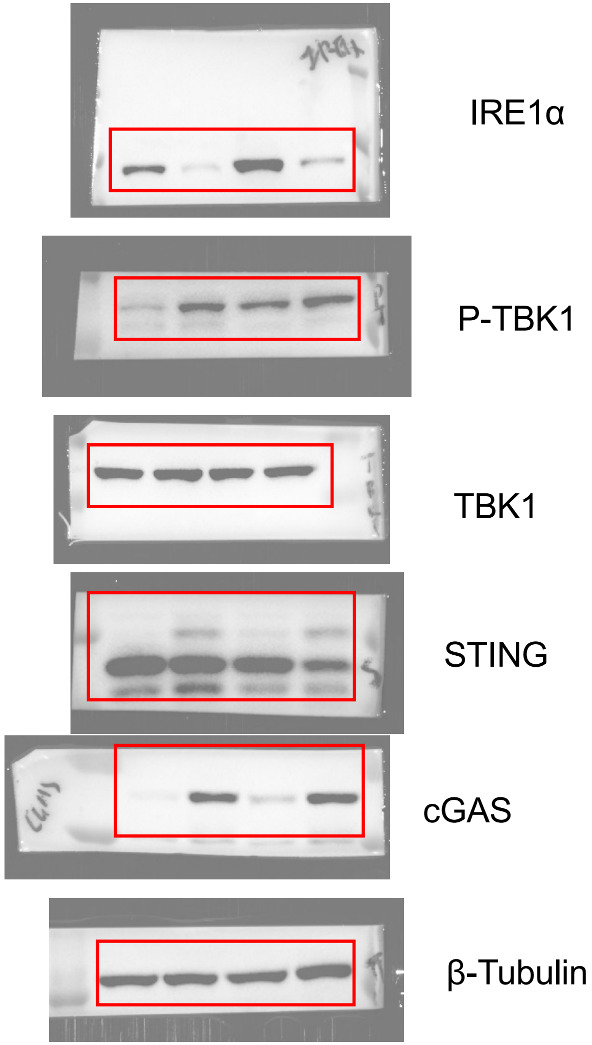

Supplement: Supplementary file 9 — Full length original western blots [file 41419_2025_7999_MOESM9_ESM.pdf]
